# Selective catalytic hydrogenation of C_2_H_2_ from plasma-driven CH_4_ coupling without extra heat: mechanistic insights from micro-kinetic modelling and reactor performance†

**DOI:** 10.1039/d4ey00203b

**Published:** 2025-01-16

**Authors:** Eduardo Morais, Fabio Cameli, Georgios D. Stefanidis, Annemie Bogaerts

**Affiliations:** a PLASMANT, Department of Chemistry, University of Antwerp Campus Drie Eiken Antwerp 2610 Belgium annemie.bogaerts@uantwerpen.be; b Laboratory for Chemical Technology, Ghent University Tech Lane Ghent Science Park 125 Ghent B-9052 Belgium georgios.stefanidis@ugent.be gstefani@mail.ntua.gr; c School of Chemical Engineering, National Technical University of Athens Iroon Polytechniou 9 15780 Athens Greece

## Abstract

We study the selective catalytic hydrogenation of C_2_H_2_, the main product from non-oxidative CH_4_ coupling in gas-phase plasmas, to C_2_H_4_, a cornerstone of the global chemical industry, by experiments and temperature-dependent micro-kinetic modelling. The model is validated against new experimental data from a nanosecond pulsed plasma reactor integrated with a downstream catalytic bed consisting of Pd/Al_2_O_3_. We explore the effects of varying Pd loadings (0.1, 0.5, and 1 wt%) on the catalyst activity and the C_2_H_4_/C_2_H_6_ product distribution. Consistent with the experimental data, our surface micro-kinetic model shows that while higher Pd loadings lower the catalyst activation temperature for C_2_H_2_ conversion, they also induce over-hydrogenation to C_2_H_6_ at lower temperatures and increase oligomerisation in the experiments, which are detrimental to the C_2_H_4_ yield. The model also elucidates reaction mechanisms and pathways across different temperature regimes, expanding our understanding of the hydrogenation process beyond the experimental range. Besides highlighting the importance of optimising the metal loading to balance C_2_H_4_ and C_2_H_6_ selectivity, our findings demonstrate the effective implementation of post-plasma catalysis using a simple catalyst bed heated by hot gas from the plasma region. This study opens possibilities for testing different plasma sources, catalysts, gas flow magnitude and patterns, and catalyst bed-to-plasma distances.

## Abstract 2

Selective C_2_H_4_ synthesis from a nanosecond pulsed CH_4_ plasma followed by catalytic C_2_H_2_ hydrogenation: micro-kinetic modelling and reactor performance.

## Graphical abstract

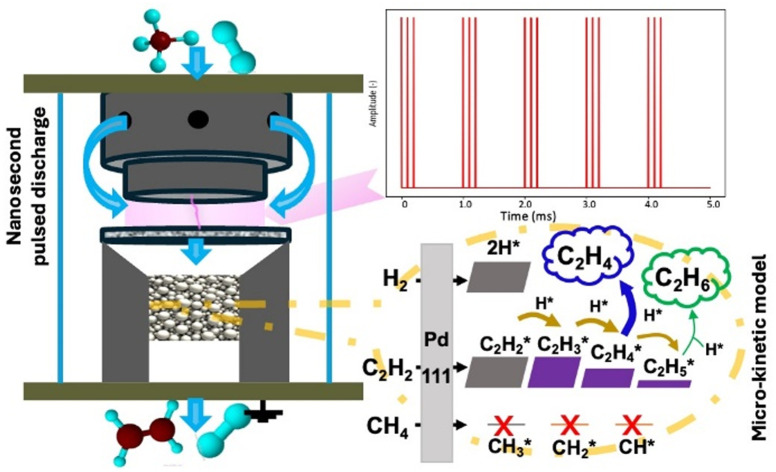

**Broader context**

The direct conversion of methane (CH_4_) to ethylene (C_2_H_4_) is thermodynamically challenging yet critical, given the demand for sustainable methods to synthesise valuable base chemicals, such as ethylene – a core molecule in global industry. The integration of plasma reactors with catalysis offers a promising solution, as it provides an efficient tool for CH_4_ coupling into C_2_H_2_, followed by a pathway to selectively steer the reaction towards C_2_H_4_. In this study, we demonstrate this synergy by catalytically hydrogenating the C_2_H_2_ plasma-product into C_2_H_4_ using Pd catalysts activated by the hot gas stream exiting the plasma reactor, without external heating. Alongside, we developed a temperature-dependent micro-kinetic surface model, providing insights into optimising C_2_H_4_ selectivity and avoiding C_2_H_6_ and oligomerisation by-products by balancing catalyst metal loading and reaction temperature. Our results broaden the understanding of coupling plasma to downstream catalysis and open new avenues for developing electrified, scalable and energy-efficient processes for ethylene synthesis and methane valorisation. These findings highlight the potential of plasma and post-plasma catalysis to play a central role in fostering a CO_2_-neutral chemical industry and promoting a more sustainable future, as well as providing a framework for further research into energy and environmental (plasma) catalysis.

## 1. Introduction

The emergence of plasma technologies for converting predominantly inert gases (such as CO_2_ and CH_4_) marks an important development within the efforts to shift the chemical synthesis of highly valuable light molecules, like C_2_H_4_ and CH_3_OH, from naphtha cracking to electrified processes with a neutral CO_2_ loop.^1,2^ The transition from traditional routes to plasma-based synthesis has positive techno-economic and environmental impacts, offering a promising solution to alleviate reliance on fossil fuels and greenhouse gas emissions.^1,3–6^ Plasma reactors are very flexible in terms of scale and targeted feedstock/products. They are also characterised by ideal coupling to (sometimes intermittent) renewable energy sources such as solar, wind, and hydroelectric power, which become increasingly widespread and cost-effective.^7,8^

While plasma-based gas conversion for chemical synthesis has continuously had tangible outcomes in recent years,^9–13^ the low product selectivity and purity is one aspect that has challenged plasma technologies in finding large-scale industrial applications. Low selectivity, often regarded as an inherent feature of applying plasma to gas conversion, is generally ascribed to the large temperature gradients observed in plasma reactors and high reactivity of plasmas.^14^ The latter results in a wide variety of reactive species (at various distinct energies), which can generate many products. For instance, in pure CH_4_ plasmas, the reported products can range from C_(s)_ to fully saturated C_3,4_ olefins, alongside H_2_.^15,16^ Distinctly, in CH_4_ conversion, the product distribution can be promptly correlated to the bulk gas temperature in the reactor, which is in turn determined by the energy density of the plasma source.^16^

On the CH_4_ pyrolysis front for C_(s)_ and H_2_ production, Fulcheri *et al.*^17^ have been leading tireless plasma research since the 1990s and in their recent work with an arc plasma (*T*_gas_ above 2000 °C), they successfully addressed this selectivity problem, attaining >90% conversion and >95% solid carbon selectivity.^9^ The developed process seems robust and has already found industrial implementation with Monolith Materials using a 1 MW pilot plasma plant to co-produce 14 000 tons of carbon black and 4600 tons of hydrogen from CH_4_ pyrolysis per year.

**Fig. 1:**
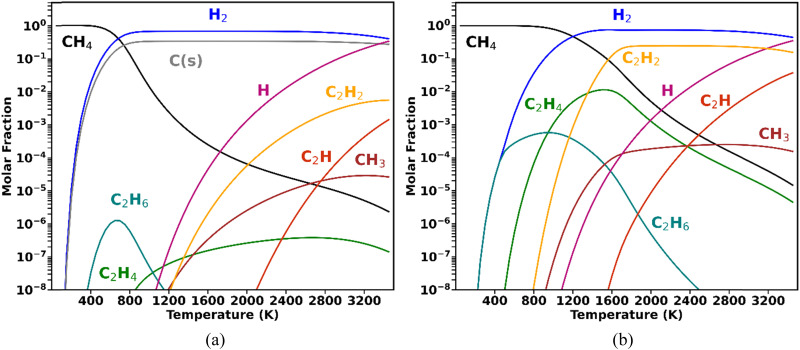
Gas-phase equilibrium composition at 1 bar, initiated with 1 mole of CH_4_, under two assumptions: (a) solid carbon is formed in equilibrium with the gas phase, or (b) there is no formation of a solid phase.

A higher degree of process control is required when CH_4_ valorisation is pursued by carbon coupling, such as non-oxidative CH_4_ coupling (NOMC), instead of cracking. Selective plasma-based synthesis of C_2_H_4_ (the most versatile light hydrocarbon, with the highest market value) at high CH_4_ conversions has not yet been accomplished. To date, the highest C_2_H_4_ selectivity from CH_4_ coupling in plasma reactors (∼50%) was reported by Delikonstantis *et al.*,^18^ with the utilisation of nanosecond pulsed discharges in a co-axial reactor with an equimolar feed of CH_4_ and H_2_. However, this level of C_2_H_4_ selectivity was only achieved at 5 bar, with C_2_H_2_ remaining the dominant product at lower pressures, as later confirmed by kinetic modelling.^19^ In fact, the attainment of high C_2_H_4_ selectivity at atmospheric pressure is impeded by the thermodynamic equilibrium of gas-phase CH_4_ shown in the diagrams in Fig. 1.

The equilibrium compositions clearly demonstrate that when CH_4_ is converted under conditions favourable to solid carbon formation (Fig. 1a), the thermodynamically favoured products are C_(s)_ and H_2_ (as in the work by Fulcheri *et al.*^9^), followed by C_2_H_2_, with negligible C_2_H_4_ production. On the other hand, plasma-driven CH_4_ pyrolysis can be performed under conditions seeking to inhibit carbon nucleation,^20^ which is illustrated for the ideal case in Fig. 1b (albeit some C_(s)_ formation is inevitable in reality). In this case, the dominant products are C_2_H_2_ and H_2_, and although formed in appreciable concentrations, C_2_H_4_ can never become the major product and its occurrence has a very narrow temperature range.^18,19^

These thermodynamic trends allow for the interpretation of common experimental findings in CH_4_ plasmas. When a plasma operates under thermal or quasi-thermal conditions, as is the case for DC arc (employed by Monolith), gliding arcs and microwave plasmas, typically the main products observed are H_2_, C_(s)_ and C_2_H_2_, with relative concentrations that depend on specific reactor configurations. Some examples can be found in ref. 9 and 21–24. Conversely, when a non-thermal plasma (such as a dielectric barrier discharge (DBD) or pulsed corona) is employed, the primary products are C_2_H_6_ (with some C_2_H_4_ generation) – mostly with low energy absorption by the gas phase, leading to poor performance in terms of CH_4_ conversion and energy efficiencies.^25,26^

Undoubtedly, this analysis reveals the essential role of catalytic C_2_H_2_ hydrogenation in plasma-based CH_4_ coupling for selective C_2_H_4_ synthesis with high conversion and competitive energy efficiency. The coupling of a nanosecond pulsed CH_4_/H_2_ plasma (with up to 40% C_2_H_2_ yield at an energy cost of 870 kJ mol^−1^)^27,28^ to post-plasma hydrogenation catalysis using a palladium-coated electrode structure has been performed by Cameli *et al.*,^29^ demonstrating a 60% overall C_2_H_4_ selectivity without external heat or further H_2_ addition. The success of this endeavour has highlighted the potential of plasma-catalyst synergy for single-pass NOMC into C_2_H_4_ in a modular fashion. Further optimisation of the structured catalyst, by employing a bimetallic Pd–Ag material, has increased the C_2_H_4_ selectivity to 76% C_2_H_4_, intensifying the process performance by lowering the downstream separation cost.^30^ Meanwhile, this approach shows great flexibility owing to the independent tuning of the plasma discharge and structured catalyst, which elicits research into different catalyst designs and compositions and alternative (perhaps simpler) catalytic setups, widening the scope of plasma-catalyst utilisation.

In this broader context, we have developed a temperature-dependent surface micro-kinetic model to investigate the selective hydrogenation of C_2_H_2_, synthesised from NOMC in a plate-to-plate nanosecond pulsed plasma reactor, using a downstream catalyst bed. The latter was packed with three different Pd/Al_2_O_3_ catalysts, with Pd loadings of 0.1, 0.5 and 1 wt%, which were activated by the heat created in the plasma. The combined (kinetic) modelling and experimental approach aims to explore the mechanisms of post-plasma C_2_H_2_ hydrogenation in the presence of unreacted CH_4_ considering the real thermal conditions in the catalyst bed downstream from the plasma zone. Building upon the current state-of-the-art,^29,30^ our objective is to extend the applicability of Cameli's post-plasma catalytic work by demonstrating how a classic catalyst bed can be utilised to harness plasma-generated heat and drive selective C_2_H_2_ hydrogenation. This strategy may open opportunities for the use of other metal catalysts in this process. Moreover, the new temperature-dependent surface micro-kinetic model aids in interpreting the reactivity results, providing insights into the adsorption/desorption mechanisms and reaction pathways that underlie the observed selectivity trends.

## 2. Experimental and computational methodology

### 2.1. Experimental setup

**Fig. 2:**
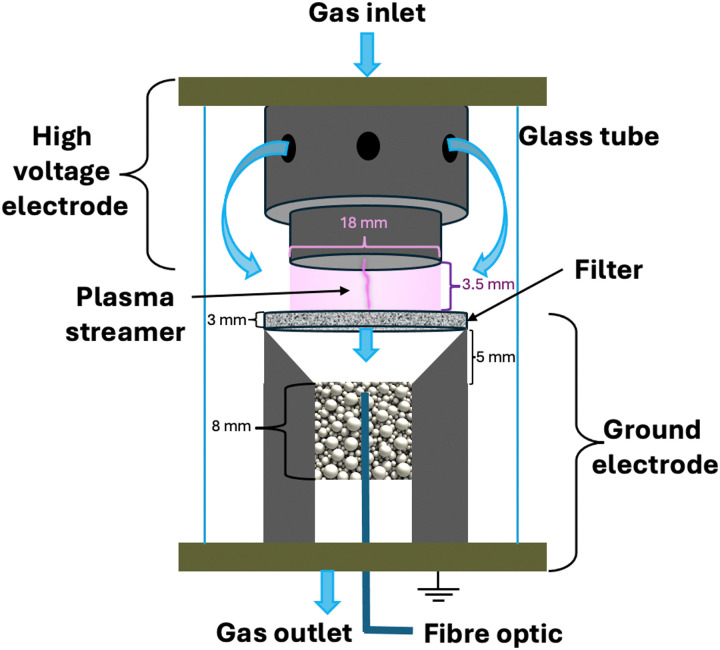
Schematic of the plate-to-plate plasma reactor with a post-plasma catalytic bed for hydrogenation. Experiments carried out with 100 sccm CH_4_ and 100 sccm H_2_, with three bursts at 10 kHz, 3000 p s^−1^, 50% voltage amplitude. The catalytic bed is composed of 200 mg Pd/Al_2_O_3_ and 800 mg Al_2_O_3_.

The reactor configuration has been described in detail in our previous work.^29^ A schematic representation of the experimental setup is reported in Fig. 2. The reactant gas consists of 100 sccm of CH_4_ and 100 sccm of H_2_ feeds (regulated by two Brooks GF40 mass flow controllers) which enter the reactor from the top of the high voltage electrode. Two parallel electrodes promote electrical breakdown of the recirculating gas. The ground electrode is composed of a 3D-printed body which hosts a stainless-steel sintered filter (AmesPore, porosity 5 μm), to prevent solid carbon deposit from entering the catalytic section downstream. Thus, the catalytic step follows the gas-phase plasma activation sequentially. An NPG-18/100k (Megaimpulse Ltd) power supply is used to ignite and sustain the nanosecond-pulsed discharge (NPD) in the plate-to-plate plasma reactor. Modulation and control of the plasma signal are attained *via* a waveform generator (Agilent 33220A) and an oscilloscope (Wavesurfer 10, Teledyne Lecroy). The same energy pattern is applied in all experiments, with 3000 pulses per second distributed in three bursts with a frequency of 10 kHz. The applied voltage amplitude is set at 50% of the maximum attainable by the power supply. A visual representation of the energy pattern scheme is reported in the ESI† (Fig. S1). The voltage (*V*) signal is used to calculate the power (*P*) dissipated in the discharge *via* a resistive coupler (RC20, Megaimpulse), which allows assessment of the forward and reflected energy (*E*) by measuring the voltage across the circuit with a fixed impedance (*Z*), as per the equation below:
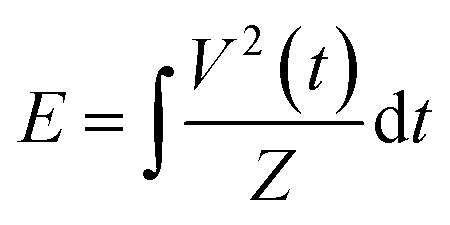


### 2.2. Catalyst preparation and characterisation

The catalysts used in the post-plasma region were produced *via* incipient wetness impregnation: a solution of Pd(NO_3_)_2_ (Alpha Aesar) was used as the catalyst precursor and added to α-Al_2_O_3_ powder (Alpha Aesar, 99.95% purity, particle size: 0.25–0.45 μm, pores volume: 0.35 mL g^−1^). Different dilutions of the original solution containing 10 wt% Pd were prepared to attain three metal loadings of 0.1, 0.5, and 1 wt% Pd/Al_2_O_3_. Overnight drying at 120 °C and calcination at 600 °C for 6 h followed the impregnation step. Metal dispersion was assessed *via* pulsed H_2_ chemisorption (Autochem II 2920, Micromeritics) of the different catalysts. The metal load is determined *via* ICP-AES (iCAP 6500, Thermo Scientific) as per the ISO 11885 methodology.

### 2.3. Downstream catalytic C_2_H_2_ hydrogenation

The post-plasma catalytic hydrogenation step is performed by packing 200 mg of catalyst, diluted in 800 mg of Al_2_O_3_, into the region downstream from the filter which serves as the ground electrode (Fig. 2). Thereby, the catalyst particles interact with the products formed *via* the recombination of plasma-generated radicals. Operando temperature monitoring was obtained through a FiSens fibre optic inserted in the catalyst bed. This fibre optic probe can measure multiple temperature points at 5 mm intervals along its axis. However, the length of the catalytic bed (8 mm) was only sufficient for two temperature measurements. The catalyst bed temperature was not independently regulated, as no external heating was applied during the experiments. Instead, the temperature was determined by two heat sources: the gas stream exiting the plasma region (*i.e.*, identical heating in all experiments, given the uniform energy profile applied in the plasma), and the exothermic hydrogenation reactions occurring at the catalyst surface. Since the contribution of hydrogenation reactions to heating varies with catalyst loading, different bed temperatures were observed across the experimental conditions. The outlet stream from the system is analysed *via* on-line gas chromatography (3000 MicroGC, Inficon), whereby a molesieve column (10 m) with backflush (3 m, Plot U) elutes H_2_, N_2_, and CH_4_, whilst a Plot U column (10 m) with backflush (1 m, Plot Q) elutes C_2_ and C_3_ species. Internal standard N_2_ is fed directly to the GC column, to calculate the total gas volume at the outlet of the reactor, owing to the changing number of moles in the plasma coupling and post-plasma catalytic hydrogenation reactions, as outlined below. This ensures that gas expansion/contraction is properly considered for accurate appraisal of conversion and selectivity.^31^2CH_4_ → C_2_H_2_ + 3H_2_C_2_H_2_ + H_2_ → C_2_H_4_ + MC_2_H_4_ + H_2_ → C_2_H_6_ + MThe composition of the gas stream entering the catalyst bed was assessed *via* GC measurements from experiments carried out in the absence of catalyst. In this case, the equations used to evaluate the CH_4_ conversion and C_2_ product selectivity can be found in ref. 29, where we focus on these plasma-alone experiments. The GC data was used to calculate the concentrations of unreacted CH_4_, and formed C_2_H_*y*_ products, which in turn were taken as a reference to isolate the contributions of plasma and catalysis in the overall conversion and selectivity from the coupled plasma-catalytic process. In this study, the more relevant metrics of C_2_H_2_ conversion and C_2_H_4_/C_2_H_6_ selectivity were calculated using the equations shown in Section 2.4(d) below, both for the experiments (*via* GC analysis of the outlet gas treated by plasma and catalysis) and the model (using calculated densities). The experimental plasma-alone concentrations were also used as input in the micro-kinetic model (initial partial pressures), as explained below.

### 2.4. Surface kinetic model

#### (a) Gas composition, species and reactions included in the model

The initial gas composition used in the model was identical to that measured at the outlet of the plasma reactor. The partial pressures of the gas were calculated and inserted in the model as follows: H_2_ = 0.528; CH_4_ = 0.300; C_2_H_2_ = 0.160; C_2_H_4_ = 0.010 and C_2_H_6_ = 0.002 (the reference pressure is 1 bar).

The species and reactions considered in the surface kinetic model are outlined in Table 1.

**Table 1:** Species included in the model (the asterisk, *, denotes an empty surface site, while adsorbed species are followed by *), reaction network with an indication of the initial, transition and final states and respective activation and reaction energies in eV

Gas-phase species		Surface species	
H_2_(g) CH_4_(g) C_2_H_2_(g)		H* C_2_H_2_* C_2_H_3_* C_2_H_4_*	
C_2_H_4_(g) C_2_H_6_(g)		C_2_H_5_* CH_3_* CH_2_* CH* C*	
Reaction	Initial state ⇌ transition state ⇌ final state	Activation energy (eV)	Reaction energy (eV)
r_1_	H_2_(g) + 2 * ⇌ *–H–H–* + * ⇌ H* + H*	0.28	−0.83
r_2_	C_2_H_2_(g) + * ⇌ C_2_H_2_–* ⇌ C_2_H_2_*	0.00	−1.67
r_3_	C_2_H_4_(g) + * ⇌ C_2_H_4_–* ⇌ C_2_H_4_*	0.00	−0.76
r_4_	C_2_H_6_(g) + 2 * ⇌ *–C_2_H_5_–H–* ⇌ C_2_H_5_* + H*	1.18	0.18
r_5_	C_2_H_6_(g) + 2 * ⇌ *–CH_3_–CH_3_–* ⇌ CH_3_* + CH_3_*	2.89	0.60
r_6_	CH_4_(g) + 2 * ⇌ *–CH_3_–H–* ⇌ CH_3_* + H*	1.29	0.31
r_7_	C_2_H_2_* + H* ⇌ *C_2_H_2_–H–* ⇌ C_2_H_3_* + *	0.95	0.05
r_8_	C_2_H_3_* + H* ⇌ *C_2_H_3_–H–* ⇌ C_2_H_4_* + *	0.64	−0.48
r_9_	C_2_H_4_* + H* ⇌ *C_2_H_4_–H–* ⇌ C_2_H_5_* + *	0.85	0.11
r_10_	C_2_H_5_* + * ⇌ *CH_3_–CH_2_–* ⇌ CH_3_* + CH_2_*	2.18	0.74
r_11_	C_2_H_4_* + * ⇌ *CH_2_–CH_2_–* ⇌ CH_2_* + CH_2_*	1.89	1.17
r_12_	C_2_H_3_* + * ⇌ *CH_2_–CH–* ⇌ CH_2_* + CH*	1.47	0.34
r_13_	C_2_H_2_* + * ⇌ *CH–CH–* ⇌ CH* + CH*	1.63	0.04
r_14_	CH_3_* + * ⇌ *CH_2_–H–* ⇌ CH_2_* + H*	1.13	0.32
r_15_	CH_2_* + * ⇌ *CH–H* ⇌ CH* + H*	0.79	−0.35
r_16_	CH* + * ⇌ *C–H* ⇌ C* + H*	1.42	0.38
r_17_	H_2_(g) + C_2_H_4_* + * ⇌ *–H–C_2_H_5_* ⇌ C_2_H_5_* + H*	1.44	−0.72

Our model is based on this reaction network and the Pd(111) energetics derived from density functional theory (DFT) calculations performed by Nørskov *et al.*^32^ These DFT calculations were carried out using Quantum Espresso, with the exchange–correlation contribution to the electronic energy approximated by the BEEF-vdW functional. We refer to the cited study for additional DFT details. The reaction network consists of adsorption and desorption of the gas-phase molecules – reactions r_1_–r_6_ (with H_2_, C_2_H_6_ and CH_4_ dissociating upon adsorption); hydrogenation and de-hydrogenation – reactions r_7_–r_9_, r_14_–r_16_; and surface dissociation and recombination – reactions r_10_–r_13_ and r_17_. In turn, the model provides quantitative information on catalyst activity and selectivity as a function of temperature. The relevant equations and formulas are presented in the following sections.

#### (b) Numerical details, governing equations and model solution

The micro-kinetic surface model was constructed using the CSTR (continuously stirred tank reactor) approach, which assumes perfect mixing throughout the simulation, with species densities and coverages being considered uniform within the reactor volume. The changes in the number density of gas-phase species (Table 1) as a function of time were calculated using the following balance equation.
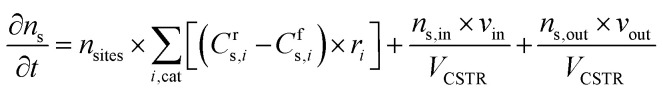
where *n*_s_ is the number density of species *s* and *t* is the simulation time. The first term on the right side is related to the change in *n*_s_ due to surface reactions at the catalyst, with *C*^r^_s,*i*_ and *C*^f^_s,*i*_ being the stoichiometric coefficients of species *s* in reaction *i* (reverse, r, and forward, f), and *r*_*i*_ being the reaction rate (expressed in s^−1^). This term must be multiplied by the total volumetric density of surface sites *n*_sites_ (in cm^−3^, with calculation details given in Section S2 in the ESI†) to have the rate involving gas species correctly expressed in cm^−3^ s^−1^. The approach utilised to calculate the rates will be presented later in this section, following the description of the balance equation for surface species. The second and third terms represent the change in number density due to gas molecules entering and exiting the reactor, respectively. In these, *n*_s,in_ is the species density at the inlet and *n*_s,out_ at the outlet (note that in a CSTR model, *n*_s,out_ equals the species density *n*_s_ in the reactor), *V*_CSTR_ is the gas volume in the reactor, and *v*_in_ and *v*_out_ are the volumetric flow rate entering and exiting the reactor, respectively.

The volumetric flow rate of the exiting gas (*v*_out_) used in the equation above is calculated as follows, so that the total pressure (*p*_total_, expressed in Pascal here for unit consistency) in the reactor is maintained constant at ambient pressure in all simulations.
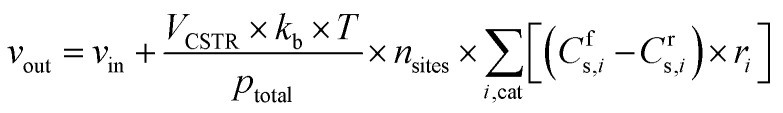
In which *k*_b_ is the Boltzmann constant and *T* is the temperature. In practice, this equates the exiting volumetric flow rate to the incoming flow rate plus the change in volume resulting from catalytic reactions.

Similar to the balance equations above for the gas-phase species, the changes in the surface coverage (*θ*_s_) of surface species *s* (Table 1) as a function of time *t* are described using the following balance equation, in which *C*^f^_s,*i*_ and *C*^r^_s,*i*_ are the stoichiometric coefficients of species *s* in reaction *i*, with the forward (f) and reverse (r) reactions, and *r*_*i*_ is the reaction rate of the surface reactions (again expressed in s^−1^).
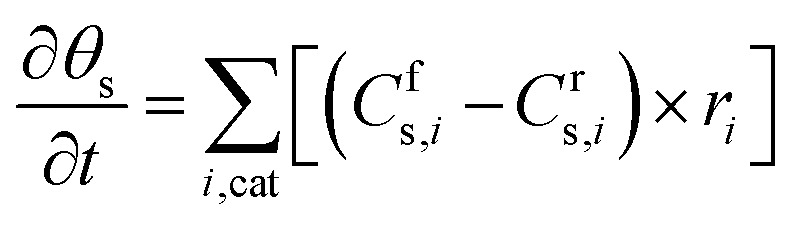
The reaction rate (*r*_*i*_) in the above equations depends on the rate coefficient, species activity and reaction stoichiometry, and is defined as follows.
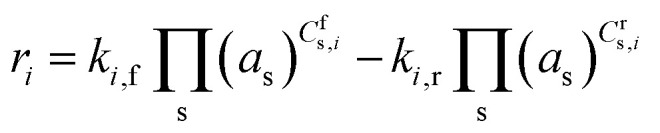
where *k*_*i*,f_ and *k*_*i*,r_ are the forward and reverse reaction rate coefficients, respectively, and *a*_s_ is the activity of species *s*. The latter may be the partial pressure of gas phase species (*p*_s_, divided by *p*_total_ to maintain *k*_*i*,f_ and *k*_*i*,r_ in units of s^−1^) in surface reactions, or the fractional coverage (*θ*_s_) for surface species. Note that number densities and partial pressures are interconvertible *via* the ideal gas law. The rate coefficients *k*_*i*,f_ and *k*_*i*,r_ are calculated using transition state theory with the following equation.
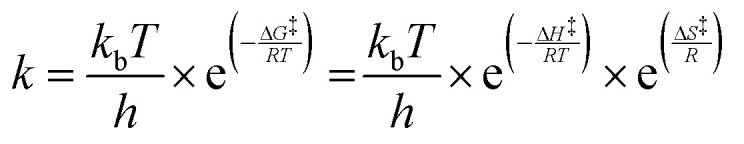
In this, *h* is Planck's constant, *R* is the universal gas constant, and Δ*G*^‡^, Δ*H*^‡^ and Δ*S*^‡^ are the differences in Gibbs free energy, enthalpy and entropy, respectively, between the initial state and the transition state (see Table 1). The inputs for enthalpy and entropy differences and their respective temperature-dependent thermodynamic corrections are discussed in the following section.

The model is solved using an in-house python code which was written for applications in post-plasma and plasma catalysis. A version of this code is quoted in Section S3 in the ESI.† The input data required to run the code include the reaction network (see Table 1), the formation enthalpies and rotational and vibrational wavenumbers of species in the model (see Table S1 in Section S4 of the ESI†), and the gas-phase species parameters for the Shomate equation (taken from the NIST database).

#### (c) Assumptions and approximations

This study concerns a pure post-plasma catalysis micro-kinetic model with only stable gaseous molecules present in the gas flow, because plasma-derived radicals rapidly recombine and are thus absent in the catalyst bed. The construction of the model is based on a CSTR approach, which assumes that the densities of the gas species and surface coverages are uniformly distributed over the reactor volume.^33,34^ The CSTR choice is intended to achieve a manageable computational complexity, whilst solving the model inexpensively for the three Pd loadings across the wide temperature range investigated in this work. The applicability of this CSTR model (over a PFR model, plug flow reactor) is also corroborated by the relatively large Péclet number (Pe) expected for packed bed systems.^35^ We believe that the PFR approach could improve the accuracy of the model's predictions by accounting for the changing concentrations across the catalyst bed. However, due to the packing of solids in the reactor utilised in the experiments (*i.e.*, non-trivial gas flow patterns and large Pe), both axial and radial gas mixings are significant, ultimately rendering the PFR approach also approximative. In a PFR configuration, the conversion would likely be higher than in our CSTR model because the latter assumes perfect mixing, thus immediately leading to lower concentrations. In turn, this causes positive order reactions to proceed at a lower rate, which yields a lower conversion for the same residence time as a PFR model.

The temperature of the catalyst bed is also assumed to be uniform throughout the reactor and the model assumes thermal equilibrium between the gas phase and catalyst surface. While temperature gradients will exist in the reactor, their assessment would require a higher-dimensional model incorporating heat transfer mechanisms and gas flow dynamics. Such analysis, though valuable, extends beyond the capabilities of our current 0D framework, which focuses on capturing the chemical kinetic behaviour of the system.

No formation of solid products at the catalyst's surfaces (polymers and carbon black) is considered in the model. Whilst very interesting, such modelling endeavour would rely on DFT data which is presently unavailable and would require a higher dimensional model, which is not within the scope of this study. With respect to the surface reactions, the model calculates rate coefficients based on transition state theory, as explained in the above section, whilst employing DFT-derived activation barriers as input in the rate expressions. While this approach yields more accurate rates than those estimated using sticking coefficients or reaction barriers,^33^ it is inherently limited by the availability and quality of DFT data. In this study, all activation energies and frequencies (used to calculate entropies and temperature-dependent corrections) were extracted from the work by Nørskov *et al.*^32^ who have described the dehydrogenation of C_2_H_6_ over many close-packed metal surfaces (see Table S1 in Section S4 of the ESI†).

**Fig. 3:**
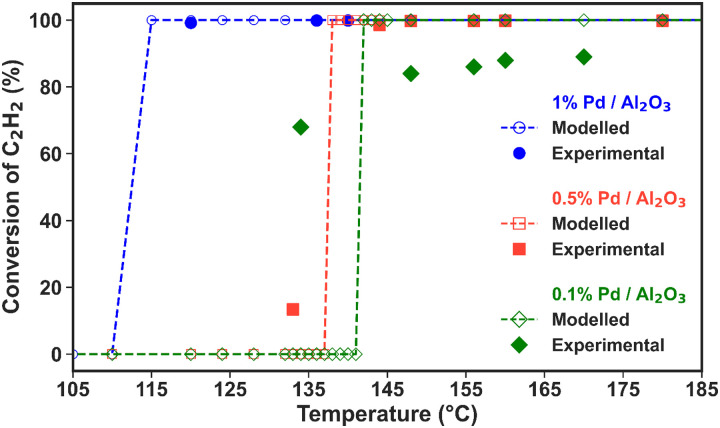
Experimental and modelled C_2_H_2_ conversion as a function of temperature (average temperature measured in the catalyst bed and assumed to be identical for solid and gas phases) for the three Pd/Al_2_O_3_ catalysts (see the legend). The number of experimental data points (solid markers) is different for each catalyst.

**Fig. 4:**
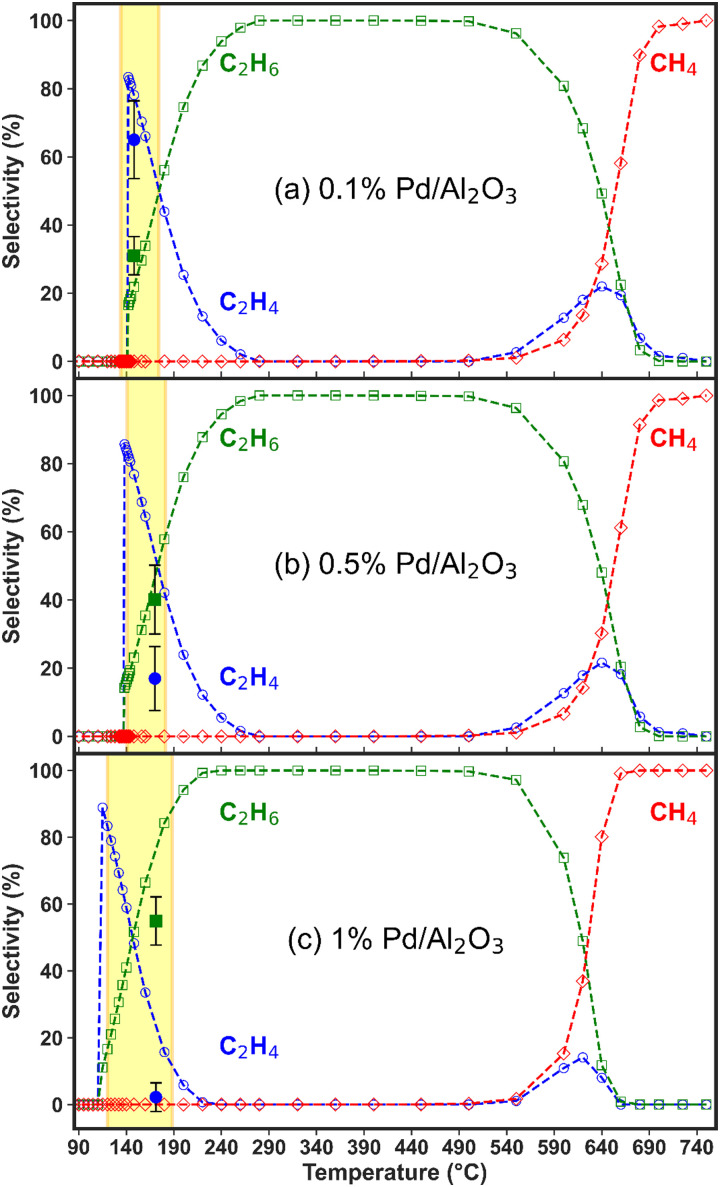
Experimental and modelled C_2_H_4_, C_2_H_6_ and CH_4_ selectivity as a function of temperature (average temperature measured in the catalyst bed and assumed to be identical for solid and gas phases) for the (a) 0.1%, (b) 0.5%, and (c) 1% Pd/Al_2_O_3_ catalysts. The solid markers correspond to the experimental selectivity measured within the temperature range highlighted by the shaded area (yellow rectangle) on the graph. The dashed lines and hollow markers show the trends predicted by the model.

To account for the activity of the Pd(100) and Pd(211) facets (which may be considerable depending on nanoparticle morphology and particle size),^36,37^ we performed a sensitivity analysis by applying the adsorption energies for C_2_H_2_ and C_2_H_4_ on Pd(100) and Pd(211) in our micro-kinetic model (see Table S2 in the ESI†). These facets were chosen based on their respective lowest and highest reported activities in the literature.^36^ Due to the lack of comprehensive DFT data for all reaction species on Pd(100) and Pd(211), we retained our original reaction network developed for Pd(111) and substituted the available adsorption energies for C_2_H_2_ and C_2_H_4_. The details of this analysis can be found in Section S5 in the ESI.† The results indicate that Pd(100) is by far the least active facet, with no observable C_2_H_2_ conversion below ∼600 °C (Fig. S2a, ESI†). Conversely, Pd(211) is an overly active surface, fully hydrogenating C_2_H_2_ to C_2_H_6_ at temperatures as low as 90 °C (Fig. S2b, ESI†). These trends, albeit inherently qualitative due to the incomplete DFT datasets, do not align with our experimental results (see Fig. 3 and 4 in Section 3). Thus, we conclude that Pd(111) is the most appropriate facet to model C_2_H_2_ hydrogenation, for the conditions under study in this work.

#### (d) Conversion and selectivity

For both model and experiments, the overall C_2_H_2_ conversion (*χ*_C_2_H_2__), and C_2_H_4_ (*S*_C_2_H_4__) and C_2_H_6_ (*S*_C_2_H_6__) selectivity, can be derived using the following equations.
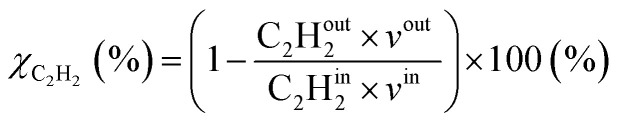

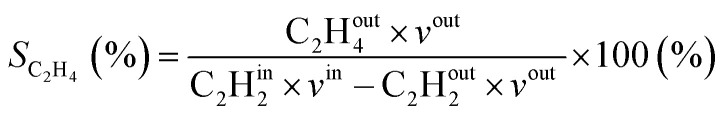

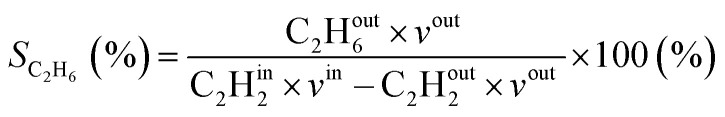
where in the experiments, C_2_H^out^_2_ represents the volume fraction of C_2_H_2_ exiting the reactor (measured by GC in the coupled plasma and catalysis experiments) and *v*^out^ is the outlet volumetric flow rate. Likewise, C_2_H^in^_2_ represents the inlet C_2_H_2_ volume fraction (reference value, measured in the absence of catalyst, as explained above) and *v*^in^ is the inlet volumetric flow rate. In the model, C_2_H^out^_2_ and *v*^out^ are the C_2_H_2_ density and volumetric flow rate at steady state in the outflow, C_2_H^in^_2_ and *v*^in^ are the initial C_2_H_2_ density and volumetric flow rate, respectively. For selectivity, C_2_H^out^_4_ and C_2_H^out^_6_ are GC-derived volume fractions of C_2_H_4_ and C_2_H_6_ in the outlet stream in the experiments, respectively; while in the model these are the densities of C_2_H_4_ and C_2_H_6_ in steady state, respectively (also corresponding to the outflow).

Additionally, the modelled densities are also used to calculate CH_4_ (S_CH_4__) selectivity using the equation below, with CH^out^_4_ being the CH_4_ density in the steady state (outflow) and CH^in^_4_ being the initial CH_4_ density.
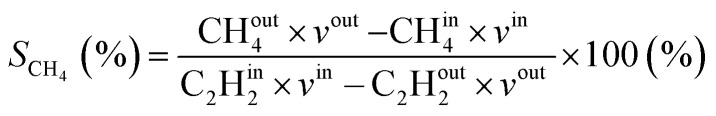
The volumetric flow rates at the inlet *v*^in^ and outlet *v*^out^ are explicitly included in these equations to account for the effect of pressure changes due to gas expansion/contraction (as explained above) and temperature increase during the reaction.^38^

## 3. Results and discussion

### 3.1. Plasma reactor for CH_4_ coupling – experimental and modelled results

The gas-phase chemistry within the NPD reactor has been thoroughly characterised in our previous studies.^19,27,29^ A comprehensive reaction mechanism has revealed that at atmospheric pressure, CH_4_ coupling predominantly produces C_2_H_2_ through the stepwise dehydrogenation of C_2_H_6_, which itself is formed *via* the recombination of CH_3_ radicals.^19,20,26,39^ Unsurprisingly, CH_3_ species are the most abundant carbon-based radicals generated from CH_4_ dissociation *via* electron-impact reactions. The principal mechanism for the coupling of electron energy to gas-phase heating involves electron-impact vibrational excitation of CH_4_ and H_2_ molecules followed by rapid vibrational–translational (VT) relaxation reactions.^40^ The gas temperature within this NPD plasma can reach up to 1500 °C in the vicinity of the discharges, which in turn promotes dehydrogenation of C_2_H_6_ to C_2_H_4_ (but also to C_2_H_5_ and C_2_H_3_ radicals) and ultimately to C_2_H_2_.^19^

As a result, this plasma configuration achieves a C_2_H_2_ selectivity of approximately 83% under the specified operating conditions (*i.e.*, three bursts at 10 kHz, 3000 pulses s^−1^). The conversion of CH_4_, co-fed with an equimolar amount of H_2_, averages around 46%. The selectivity for C_2_H_4_ and C_2_H_6_ is about 5% and 1%, respectively. The remaining percentage is attributed to small quantities of unquantified hydrocarbon products (*e.g.*, C_3_ and C_4_ species) and solid carbon, which amounts to about 3% of the converted CH_4_ in weight.

### 3.2. Post-plasma catalytic hydrogenation of C_2_H_2_ to C_2_H_4_

The high C_2_H_2_ selectivity attained in this NPD reactor enables effective tandem hydrogenation to C_2_H_4_, which is catalysed by the Pd/Al_2_O_3_ material placed downstream from the plasma discharge. This strategy allows for the flexible adjustment of the product distribution in a modular fashion, which can be tailored to meet oscillatory market demands. Notably, the catalytic hydrogenation reaction is solely activated by the hot gas flowing from the discharge zone, and it is self-sustained by its exothermic nature (*i.e.*, C_2_H_2_ + H_2_ → C_2_H_4_, with Δ*H*^0^ = +175.9 kJ mol^−1^; and C_2_H_2_ + 2H_2_ → C_2_H_6_, with Δ*H*^0^ = +311.5 kJ mol^−1^). However, the three Pd loadings within the Pd/Al_2_O_3_ catalyst, along with the dispersion degree of the active metal, promote varying C_2_H_2_ conversion and C_2_H_4_/C_2_H_6_ product distribution trends within different temperature ranges. Consequently, both the reaction temperatures and kinetics may differ with the catalyst used. In the subsequent sections, we discuss the reactivity results for each catalyst within the temperature window measured in the experiments and apply our model to extrapolate the selectivity behaviour at higher and lower temperatures. Whenever possible, we compare the model predictions with our experimental data to reinforce the credibility of these temperature extrapolations.

#### (a) C_2_H_2_ conversion

Both experimental and modelled results indicate that the reactivity of the Pd/Al_2_O_3_ catalysts is influenced by the Pd loading and the dispersion degree of Pd atoms within the material. As seen in Fig. 3, the catalysts with higher Pd loading (and greater metal dispersion) exhibit a lower temperature threshold for activation, *i.e.*, initiation of C_2_H_2_ hydrogenation. For the 1% Pd catalyst (with a dispersion of 10%), the onset of C_2_H_2_ conversion occurs between 110 and 120 °C in the experiments. However, this activation temperature range increases to 130–137 °C and 135–144 °C as the Pd loading decreases to 0.5% and 0.1%, with 7% and 33% dispersion, respectively. Note that the dispersion is not determined by the loading, but only related to the preparation method. The lower C_2_H_2_ conversion onsets observed for the higher Pd loadings can be attributed to the enhanced rates of C_2_H_2_ and H_2_ adsorption due to larger amounts of active sites. In turn, these higher rates give rise to more intense surface heating of the catalysts with higher loading (due to the exothermic nature of C_2_H_2_ hydrogenation), further accelerating the reaction rates and conversion. These effects are determined by both higher loading and higher metal dispersion, which decrease the activation temperature threshold, explaining the similar activity of the 0.5 and 0.1% loadings.

Fig. 3 also shows that the modelled results align well with the experimental data points in the region where the temperature is exclusively dictated by the plasma discharge and the exothermic heat from the hydrogenation reaction. The lack of control over the experimental temperature does not allow mapping of the catalyst activity across different temperatures in these experiments. However, these results prove that the post-plasma catalytic setup is suitable for C_2_H_2_ hydrogenation, even at low metal loading, as all catalysts attain C_2_H_2_ conversion above 90% under the experimental conditions at the steady state. The modelled trends confirm the catalysts’ high activity for C_2_H_2_ hydrogenation and can give an indication of the behaviour at higher temperatures. The modelled C_2_H_2_ conversion trends in the 80 to 750 °C temperature range can be found in Fig. S3 in the ESI† (Section S6, ESI†).

Nonetheless, the model underestimates C_2_H_2_ conversion at lower temperature for the 0.1% Pd/Al_2_O_3_ and 0.5% Pd/Al_2_O_3_ catalysts, and it suggests a sharp transition from nil to complete conversion at 137 and 142 °C, whilst experimental data show a more gradual increase. These discrepancies can be ascribed to the temperature input used in the model. The model relies on the gas temperature at the catalyst active sites, while in the experiments, the fibre optic temperature sensor is positioned along the axial axis of the catalytic bed; and it is reasonable to assume that the catalyst surface may be warmer than the surrounding gas due to the exothermic reactions occurring at the active sites. Indeed, it would be very insightful to investigate the heat transfer from the warmer catalyst surface to the gas phase and the temperature gradient in the reactor, as well as the impact of the exothermic chemical reactions, by solving an energy balance equation. However, the current model is unable to capture these effects, as the dynamics of heat transfer and gas flow cannot be accurately considered in this zero-dimensional model. While this is outside the scope of this study, in our future work, we plan the construction and application of a dedicated higher-dimensional computational fluid dynamics and surface kinetics model to explore these aspects as well.

#### (b) Product selectivity

We have applied the model to calculate the product selectivity from C_2_H_2_ hydrogenation over a wide range of temperature (up to 750 °C), as depicted in Fig. 4(a)–(c). Within the temperature range registered in the experiments (indicated by the yellow rectangles in Fig. 4), the model predictions agree well with the experimental results (obtained as a single data point for each set of catalytic material) for the 0.1% Pd catalyst (Fig. 4a). This agreement is however less accurate for the other two catalysts. At lower temperatures, the model predicts that C_2_H_4_ is the main product when the reaction begins, as it is thermodynamically favoured over further hydrogenations to C_2_H_6_ (a mechanistic analysis is provided in the following section). Over the 0.1% Pd catalyst, the calculated C_2_H_4_ selectivity peaks at 83% (with 17% C_2_H_6_ selectivity) at 142 °C, *i.e.*, the onset of the activation temperature, while the experimental values are 65% and 31%, respectively (though with relatively large error bars). Furthermore, the model suggests an inversion in C_2_H_4_ and C_2_H_6_ selectivity as the temperature increases, with C_2_H_6_ becoming dominant due to prompt over-hydrogenation of C_2_H_4_. However, this could not be validated experimentally because no external catalyst heating was applied in the experiments; hence only one data point is available, corresponding to the temperature reached by plasma heating (and the exothermic hydrogenation reactions).

All catalysts attain near-complete C_2_H_2_ conversion in the experiments (90% for 0.1% Pd/Al_2_O_3_ and >99% for 0.5 and 1% Pd/Al_2_O_3_, see Fig. 3), which is advantageous for downstream separation steps. Thus, under these conditions, the Pd loading is the only independent variable controlling the C_2_H_4_ selectivity. Notably, the highest C_2_H_4_ yield (*i.e.*, 12%, at 142 °C) is achieved by 0.1% Pd/Al_2_O_3_, which favours higher C_2_H_4_ selectivity despite a slightly lower C_2_H_2_ conversion.

The C_2_H_4_ yield is the major metric of successful CH_4_ non-oxidative coupling, as it is the most valuable product. Its maximum value is determined by the catalytic C_2_H_2_ conversion and the corresponding selectivity for C_2_H_4_, since CH_4_ conversion is driven solely by the plasma discharge. While the latter also affects the C_2_H_4_ yield, it does not vary with the studied Pd loadings, as the amount of C_2_H_2_ produced in the plasma region remains constant across the different Pd loadings in the post-plasma region. Similarly, the temperature of the gas exiting the plasma zone and entering the catalytic bed is identical in all experiments, as it is purely controlled by the plasma energy (uniform in all experiments). Our reactor configuration, where the catalytic bed is integrated in the post-plasma region without external heating, creates an inherent coupling between reaction temperature and catalyst loading. While the temperature of the gas exiting the plasma zone is constant across all experiments, the bed temperature varies with Pd loading due to the exothermic hydrogenation reactions. Although experiments at lower catalyst loadings could be useful to investigate incomplete conversion regimes, we focus here on conditions achieving full C_2_H_2_ conversion, which is critical for maximising C_2_H_4_ yield in industrial applications.

The modelled trend of decreasing C_2_H_4_ selectivity and increasing C_2_H_6_ selectivity upon rising temperature is qualitatively consistent for all three catalysts, with the key difference being the temperature at which C_2_H_6_ becomes the dominant product, see Fig. 4(a)–(c). This shift occurs at lower temperatures with increasing metal loading, indicating higher catalyst activity, and is detrimental for the desired overall C_2_H_4_ selectivity. Beyond this point, C_2_H_6_ selectivity becomes 100% due to C_2_H_4_ over-hydrogenation, until the temperature reaches about 500 °C, where another shift in reactivity occurs, leading to rapidly rising production of CH_4_ and a minor region of C_2_H_4_ formation. The thermodynamic mechanisms driving these observations are discussed in detail in the following section.

However, these higher temperatures exceed the operational range of the catalyst bed, which is limited by the temperature of the gas exiting the discharge region and the exothermic heat of the reaction. The experimental operating window of the catalyst bed (highlighted by the yellow areas in Fig. 4) corresponds to temperatures recorded over the entire hydrogenation experiment and correlate with the gas composition measured at the same acquisition time. The fast reaction kinetics results in a quick temperature rise and onset of steady-state conditions (within 10 min of plasma ignition), which does not allow the GC analysis to capture the transient composition in detail, as shown in Fig. 3. No substantial temperature increase is observed after reaching the steady-state, and all highlighted areas correspond to a relatively narrow temperature range. Given the scale difference between the modelled and experimental temperature ranges, the selectivity data from the experiments would largely overlap. For that reason, we only report one experimental point for the selectivity, as representative of the steady-state conditions.

In the experiments with the 0.1% Pd/Al_2_O_3_ catalyst, 65% C_2_H_4_ selectivity was achieved from C_2_H_2_ hydrogenation, compared to 31% C_2_H_6_ selectivity, between 140 and 175 °C (Fig. 4a), as also mentioned above. This is consistent with our previous results and other C_2_H_2_ hydrogenation reports in the literature.^29,41–44^ However, this state is reached within 5 min of plasma ignition and changes over longer periods, as the catalyst bed temperature rises to ∼170 °C and oligomerisation products begin to form from C_2_H_2_ conversion. This is accompanied by a drastic decline in C_2_ product detection in the experiments. As previously explained, oligomerisation reactions leading to solid products are not included in the model, which focuses instead on the kinetics of gaseous H_2_, C_2_H_2_, C_2_H_4_, C_2_H_6_, and CH_4_ (besides the short-lived surface species).

Nonetheless, the formation of oligomerisation by-products is the primary reason for the reduced C_2_ selectivity observed experimentally at higher temperatures. On this note, the carbon balance in the system drops from 91% at the start of the hydrogenation processes (at the lower end of the temperature window) to less than 70% after about 50 minutes, when the temperature of the gas-phase is expected to exceed 200 °C. Whilst these solid deposits on the catalyst surface may affect the activity, the time-on-stream data of the product gas composition shows a relatively constant trend over approximately 40 minutes (see Fig. S4 in the ESI†), suggesting that no major catalyst deactivation (*via* sintering, for instance) occurs.

As observed in Fig. 4(b) and (c), for both higher Pd catalyst loadings, C_2_H_6_ was detected as the primary product in the experiments, immediately after the reaction began. In fact, practically no C_2_H_4_ was detected when the 1% Pd/Al_2_O_3_ catalyst was tested, with C_2_H_6_ emerging as the sole product at ∼60% selectivity. These results are consistent with model predictions (though only at somewhat higher temperatures for the C_2_H_4_ selectivity), which suggest that C_2_H_4_ hydrogenation to C_2_H_6_ occurs at lower temperatures upon increasing Pd loading. In summary, the temperature range where C_2_H_4_ is the dominant hydrogenation product shifts to lower temperatures and narrows as the Pd loading is increased. For both catalysts, the experimental and modelled results show close alignment in terms of C_2_H_6_ selectivity, while the C_2_H_4_ selectivity is overestimated by the model in the low-temperature regime: below 240 °C for the 0.5% Pd/Al_2_O_3_ catalyst and below 210 °C for 1% Pd/Al_2_O_3_.

This discrepancy may be partially related to the difference in temperature considerations – the surface temperature input in the model *versus* bulk bed temperature measured in experiments, as explained above. However, the most likely factor contributing to selectivity disagreement is the extensive formation of oligomeric carbonaceous deposits (green oil)^36^ at full C_2_H_2_ conversion and temperatures above 170 °C, facilitated by the higher Pd loadings. Evidence supporting this mechanism is found in the calculated carbon balance of ∼76% in the experiments with both 0.5% and 1% Pd catalysts, while the hydrogen balance is greater than 91%. This indicates the formation of species with high C/H ratios, typical of oligomerisation compounds, which are not included in our model.

Additionally, an approximative evaluation of the potential impact of carbon deposition in the form of C_(s)_ was conducted in the model by analysing the CH* + * → C* + H* reaction rate (r_16_). The results show that the rate of this reaction is relatively negligible across the temperature range investigated in this study (see Fig. S5 in Section S8 of the ESI†). This suggests that deposition of C_(s)_ particles is very unlikely and cannot cause the observed differences between model predictions and the experimental data in this study. Instead, the analysis indicates that the dominant pathway is the sequential hydrogenation of CH_*x*_ surface species (see Section 4), which are converted to CH_4_ above 500 °C (rather than dehydrogenated into solid carbon).

Also noteworthy, the formation of Pd carbide during C_2_H_2_ hydrogenation is closely associated with the deposition of carbonaceous oligomers on the catalyst surface.^44^ While the present model is not able to explicitly account for PdC_*x*_ phases, their inclusion could, on the one hand, alter the Pd energetics of the surface reactions, leading to shifts in the model predictions. On the other hand, the model may see accumulation of PdC_*x*_ species on the Pd surface, leading to lower C_2_H_4_ and (especially) C_2_H_6_ product selectivity and in turn smaller discrepancies between model and experiment. However, a detailed treatment of Pd carbide effects would require dedicated DFT data (which is not available, to the best of our knowledge) and potentially higher-dimensional modelling, beyond the scope of this study, but it represents an important direction for future research. Nevertheless, as discussed, we believe the deposition of high molecular weight oligomerisation solids on the catalyst remains the primary contributor to the disagreement in selectivity.

Finally, the morphology and size of the synthesised particles should be consistent across all tested catalysts (0.1%, 0.5%, and 1% Pd/Al_2_O_3_), as these were prepared *via* the same wetness impregnation method. Moreover, identical Al_2_O_3_ support particles and dilution beads were used in all experiments, further ensuring uniformity. SEM images of the spent catalysts (Fig. S6 in Section S9 of the ESI†) confirm no observable differences in particle size or morphology, indicating that these factors are not the reason for the varying catalytic performances across the three investigated Pd loadings.

Essentially, these results highlight the dual impact of higher Pd loadings: (i) they promote prompt over-hydrogenation to C_2_H_6_ by lowering the temperature at which sequential surface hydrogenation reactions occur (namely from C_2_H_2_* to C_2_H_3_* to C_2_H_4_* to C_2_H_5_* and finally C_2_H_6_; see reactions r_7_, r_8_, r_9_ and reverse of r_4_ in Table 1), and (ii) they facilitate oligomerisation reactions at lower temperatures. Therefore, optimisation of the Pd loading in these catalysts is crucial for successful coupling between the plasma and catalysis reactors, as it significantly influences the C_2_H_4_/C_2_H_6_ product distribution and the formation of unwanted solid by-products.

#### (c) Modelled Pd reactivity and reaction pathway analysis

**Fig. 5:**
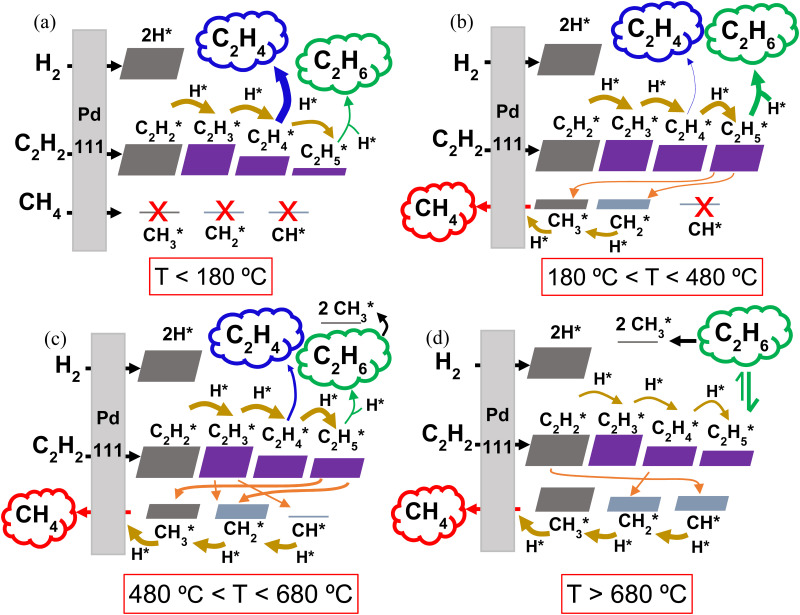
Reaction pathway diagrams illustrating the reactions between the gaseous reactants (C_2_H_2_, H_2_ and CH_4_) and the catalyst surface (a) below 180 °C, (b) between 180 and 480 °C, (c) between 480 and 680 °C and (d) above 680 °C. The resulting adsorbed surface species (marked with *) and their reactions with other surface species are also shown. The eventual desorption of the C_2_H_4_ (blue), C_2_H_6_ (green) and CH_4_ (red) products (with varying concentrations depending on the temperature) is indicated by the cloud-shaped text boxes. Adsorption reactions are indicated by black arrows, surface hydrogenation reactions by mustard arrows, and surface dissociation reactions by orange arrows. The thickness of the arrows and the size of the blocks are qualitatively representative of the reaction rates and species surface coverage, respectively.

The model was applied to evaluate the forward and reverse rates of the considered reactions as a function of time and temperature, allowing for a mechanistic analysis of the catalyst reactivity. In turn, this provides insights into the reaction pathways that dictate the observed selectivity trends. The results of this analysis are presented in Fig. 5(a)–(d), highlighting the reactivity across the low- and mid-temperature regimes and high-temperature regime, respectively. Additionally, the reaction rates relevant to the following discussions are plotted in Fig. S7–S11 (ESI†) (see Section S10 of the ESI†).

Within the studied temperature range, the adsorption of both H_2_ (into 2H*) and C_2_H_2_ (into C_2_H_2_*), onto the Pd surface occurs very rapidly, with rates approaching 10^25^ s^−1^. Following the formation of C_2_H_2_* and H*, sequential surface hydrogenation reactions (r_7_, r_8_ and r_9_ in Table 1) proceed, generating C_2_H_3_*, C_2_H_4_* and C_2_H_5_* species – with varying surface coverage, depending on the temperature (as shown in Fig. 5). At temperatures below 180 °C (Fig. 5a), desorption of C_2_H_4_* (reverse of reaction r_3_) is preferred, leading to the evolution of C_2_H_4_(g) over further hydrogenation to C_2_H_5_* (r_9_). This preference results in the predominance of the desired C_2_H_4_ product at low temperatures, aligning with the selectivity results observed in our experiments with the 0.1% Pd/Al_2_O_3_ catalyst. The desorption of C_2_H_4_* into C_2_H_4_(g) peaks at 144 °C, coincide with the highest C_2_H_4_ selectivity. In this temperature range, the coverage of C1 surface species remains negligible, as dissociative desorption of CH_4_ (r_6_) does not occur.

As the temperature rises, the rate of C_2_H_4_* desorption decreases, whilst the rates of C_2_H_4_* hydrogenation to C_2_H_5_* and subsequent hydrogenation to C_2_H_6_(g) (r_4_) increase significantly (Fig. S7 and Fig. 5b, ESI†). This behaviour was also observed by Wang *et al.*^43^ and Shi *et al.*^44^ for C_2_H_2_ hydrogenation over Cu and Au-based catalysts. As a result, the C_2_H_6_ product sees a rise in selectivity and it becomes the main product from ∼180 °C.

As the temperature is further increased, the desorption rate of C_2_H_4_* falls sharply, dropping to approximately zero around 280 °C. Simultaneously, the hydrogenation reactions to C_2_H_5_* and C_2_H_6_(g) accelerate, making C_2_H_6_ the only product seen by the model between 280 and 450 °C. These rates are plotted in Fig. S8 (ESI†). However, once the temperature reaches 450 °C, the dissociation of C_2_H_5_* into CH_3_* and CH_2_* (r_10_) begins to occur, as shown in Fig. 5b, competing with the hydrogenation to C_2_H_6_. In turn, this gives rise to the production of CH_4_(g), as CH_3_* undergoes surface hydrogenation (reverse of the r_6_ reaction, as detailed in Fig. S9, ESI†). Thus, at temperatures above 450 °C, CH_4_ becomes a product of C_2_H_2_ hydrogenation over this Pd catalyst, with its selectivity rising rapidly with temperature (*cf.*Fig. 4 above).

Interestingly, for all three Pd loadings, the model predicts the reappearance of C_2_H_4_ as a product between ∼500 and 710 °C, depending on the Pd loading (see Fig. 4 above), with a local maximum in C_2_H_4_ selectivity around 600 °C. This trend is corroborated by our rate analysis, which indicates a second region of C_2_H_4_* desorption into C_2_H_4_(g) (reverse of r_3_) within this temperature range (see Fig. S10 and Fig. 5c, ESI†). Concurrently, the dissociation rate of C_2_H_5_* into CH_3_* and CH_2_* (r_10_) steadily rises from 500 °C, and C_2_H_3_* also begins to undergo surface dissociation into CH_2_* and CH* (r_12_) (see Fig. 5c). These dissociation processes explain the sharp decline in C_2_H_6_ selectivity at elevated temperatures (*cf.*Fig. 4), as they compete with the hydrogenation steps required to form the C_2_H_4_ and C_2_H_6_ products. Indeed, the reaction rates of both C_2_H_4_* and C_2_H_5_* + H* (r_9_ and r_4_) dwindle with increasing temperature (Fig. S10, ESI†). Besides, the observed waning C_2_H_6_ production can also be ascribed to dissociative adsorption of C_2_H_6_(g) into 2CH_3_* (reverse of r_5_) (*cf.*Fig. 5c), which becomes significant from 600 °C onwards. Collectively, these reactions contribute to enhancing the catalyst surface coverage with CH_x_* species, ultimately resulting in the evolution of CH_4_(g) – which becomes the dominant product above ∼650 °C.

Beyond 680 °C, additional dissociation reactions begin to take place alongside those discussed above. These are the dissociation of C_2_H_2_* into 2CH* (r_13_), C_2_H_4_* into 2CH_2_* (r_11_) and C_2_H_6_(g) into C_2_H_5_* and H* (reverse of r_4_), as illustrated in Fig. 5d, with rates shown in Fig. S11 (ESI†). At the same time, all C_2_H_y_* hydrogenations become slower, while the hydrogenation rates of CH* (reverse of r_15_) and CH_2_* steadily increase (*cf.* thickness of the arrows in Fig. 5d). As a result, the formation of C_2_ hydrocarbons is further weakened, becoming negligible above 700 °C. Meanwhile, the CH_4_ production peaks, approaching 100% selectivity at 750 °C and explaining the trend in Fig. 4.

## 4. Conclusions

In this work, we developed a surface micro-kinetic model that simulates the catalytic C_2_H_2_ hydrogenation to C_2_H_4_, following plasma-based non-oxidative CH_4_ coupling. We validated the model against experiments using an NPD plasma reactor chemically and thermally integrated with a downstream catalyst bed, employing different Pd/Al_2_O_3_ materials with Pd loadings of 0.1%, 0.5% and 1%. All three catalysts effectively convert the C_2_H_2_ present in the exiting gas stream from the plasma discharge without external heating, with conversions above 90%. By applying fixed plasma conditions, we attain a consistent CH_4_ conversion around 46%, and C_2_H_2_ selectivity around 83% for the plasma stage, and thus post-plasma catalytic C_2_H_2_ hydrogenation into C_2_H_4_ is the main driver of the final C_2_H_4_ yield. Our experiments show that only the 0.1% Pd/Al_2_O_3_ catalyst evolves C_2_H_4_ as the main hydrogenation product, while at higher Pd loadings, C_2_H_6_ is predominantly produced. Our model predicts a slightly different trend, with higher C_2_H_4_ selectivity for all catalysts in the low-temperature regime, though C_2_H_6_ rapidly becomes predominant above 150–180 °C. The highest C_2_H_4_ selectivity is obtained at the lowest simulated temperature (*i.e.*, 142 °C, 135 °C and 115 °C for the 0.1%, 0.5% and 1% Pd/Al_2_O_3_ catalysts, respectively). In the experiments, other oligomerisation by-products are also observed, but they are not included in the model, which might explain the observed discrepancy.

Our results also show that the exothermic nature of the hydrogenation reactions induces a temperature rise within the catalyst bed, which is detrimental to the C_2_H_4_ selectivity. This is in line with the modelled reaction mechanism, which reveals that by increasing the catalyst temperature, the C_2_H_4_* desorption rate is lowered, while further hydrogenation is favoured. As this effect is highly undesirable, the deployment of more selective catalysts (*e.g.*, mixed metal alloys)^45,46^ able to fine-tune these rates may enhance the process performance.

Other key factors may influence the coupling between the plasma reactor and the catalyst bed. We believe these include the type of catalyst and support (*i.e.*, oxides *versus* metal-based), the plasma source, reactor geometry, the magnitude and type of gas flow, type of filter, and the distance between the bed and discharge region. Ultimately, these factors directly impact heat transfer, and in turn the catalyst temperature, which, as shown in this work, significantly affects the selective synthesis of C_2_H_4_. Tailoring these variables allows for fine control over the catalyst bed temperature, thereby maximising C_2_H_4_ selectivity (and yield) and catalyst performance. This research opens avenues for further exploration of coupling different plasma sources and reactors with simple post-plasma catalytic setups. Potentially, the flexibility in adjusting the catalyst bed temperature through the aforementioned factors may allow for the use of cheaper and more abundant catalysts (such as Cu and Ni), which generally require higher activation temperatures.

## Data availability

The data supporting this article have been included as part of the ESI.†

## Conflicts of interest

There are no conflicts to declare.

## Supplementary Material

EY-003-D4EY00203B-s001
