# Supplementary material for: Selective catalytic hydrogenation of C2H2 from plasma-driven CH4 coupling without extra heat: mechanistic insights from micro-kinetic modelling and reactor performance
Source: EES Catal. 2025 Jan 16;3(3):475–87. doi: 10.1039/d4ey00203b (PMC11775647; doi:10.1039/d4ey00203b)
Supplement: EY-003-D4EY00203B-s001 [file EY-003-D4EY00203B-s001.pdf]

Supplementary Information (SI) for EES Catalysis.
This journal is © The Royal Society of Chemistry 2025

Selective catalytic hydrogenation of C_2H_2 from plasma-driven CH_4 coupling without extra heat: mechanistic insights from micro-kinetic modelling and reactor performance

Eduardo Morais,[a†] Fabio Cameli,[b,c†] Georgios D. Stefanidis[b,c*] and Annemie Bogaerts[a*]

[a] *PLASMANT, Department of Chemistry, University of Antwerp, Campus Drie Eiken, Antwerp 2610, Belgium*
[b] *Laboratory for Chemical Technology, Ghent University; Tech Lane Ghent Science Park 125, Ghent, B-9052, Belgium*
[c] *School of Chemical Engineering, National Technical University of Athens, Iroon Polytechniou 9, 15780, Athens, Greece*

† Shared first authors
* Correspondence to:
Annemie Bogaerts: annemie.bogaerts@uantwerpen.be
Georgios Stefanidis: georgios.stefanidis@ugent.be and gstefani@mail.ntua.gr

Supporting Information

S1. Energy pattern

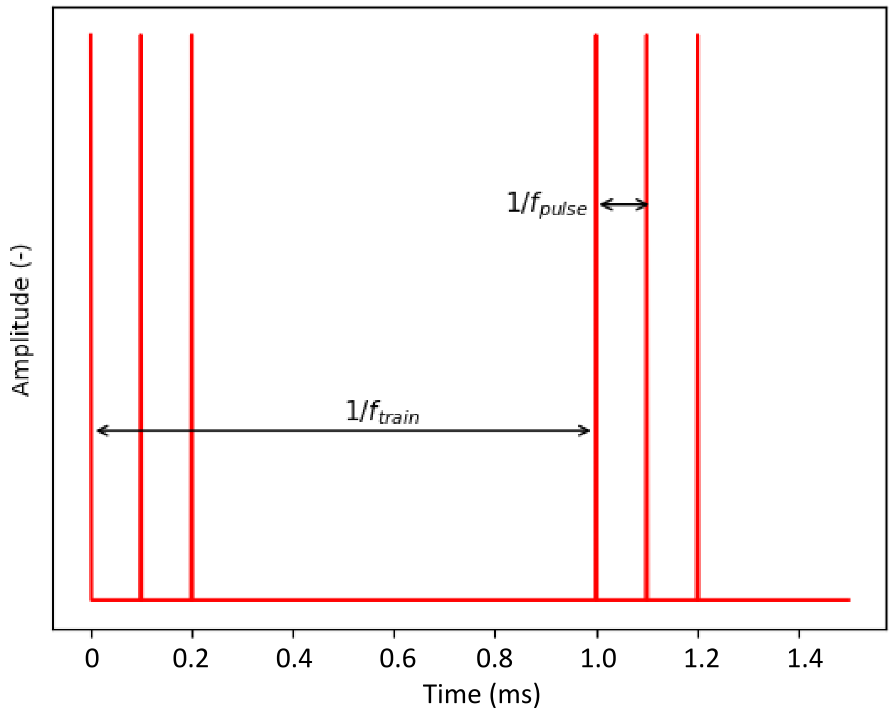

Figure S1. Visualisation of the energy pattern employed for the plasma experiments. A train of three pulses with pulse frequency of 10 kHz is imposed, with the number of pulses per second set at 3000.

S2. Calculation of number of active sites

The number of active sites can be estimated using the dispersion coefficient of the active metal particles (measured experimentally) and the total mass of the catalyst. The dispersion coefficient is the fraction of atoms exposed at the surface and is equal to the ration between the number of surface atoms and the total number of atoms of the material. Below, the calculation of the active sites for the 0.1% Pd catalyst with a dispersion coefficient of 33% is shown. This calculation is also based on the Pd atomic weight (106,42 g/mol) and mass of Pd used in the experiments, which is equal to 0.1% of 200 mg of packing material.

$$m_{Pd} = 0.1\% * 200\,mg = 2 * 10^{-4}\,g$$

$$n_{Pd} = \frac{m_{Pd}}{M_{Pd}} = \frac{2 * 10^{-4}\,g}{106.42\frac{g}{mol}} = 1.88 * 10^{-6}\,mol$$

$$\#_{Pd} = n_{Pd} * N_A = 1.88 * 10^{-6} mol * 6{,}022 * \frac{10^{23} atoms}{mol} = 1.13 * 10^{18}\,atoms$$

$$\#_{active\ sites} = \#_{Pd} * D = 1.13 * 10^{18} atoms * 33\% = 3.77 * 10^{17} active\ sites$$

S3. Solving the model

A version of the in-house python script which was used to solve the model is quoted below.

```
import re
import sys
import os
import numpy as np
from ase.symbols import string2symbols
from ase.thermochemistry import HarmonicThermo
from scipy.integrate import solve_ivp
from copy import deepcopy
import matplotlib.pyplot as plt

# Constants
N_A = 6.02214076e23
kb = 1.380649e-23

def load_data(filename, separator):
    """Loads data from a file into a dictionary."""
    with open(filename, 'r') as f:
        data = [line.strip() for line in f if line.strip()]
    labels = data[0].split(separator)
    values = [line.split(separator) for line in data[1:]]
    return {label: [value[i] for value in values] for i, label in enumerate(labels)}

def get_surf_reactions(rxn_file, site_types, surf_elements):
    """Extracts surface reactions from a file and validates them."""
    rxn_list = [re.sub(r'\s|\'|,|_g|\[|\]', '', rxn.replace('<->', '->')) for rxn in
open(rxn_file)]
    for rxn in rxn_list:
        sections = [s.split('+') for s in rxn.split('->')]
        # Check site and atom balance
        for site in site_types:
            if not all(s.count(site) == s.count(site) for s in sections):
                sys.exit(f'Site {site} is not balanced in reaction {rxn}')
        for atom in surf_elements:
            if not all(string2symbols(s).count(atom) == string2symbols(s).count(atom) for s in
sections):
                sys.exit(f'Atom balance incorrect for {atom} in reaction {rxn}')
    return rxn_list

def get_species(rxn_list, site_types):
    """Extracts gas, surface, and adsorbed species from reaction list."""
    gas_spec, surf_spec, ads_spec = [], [], []
    for rxn in rxn_list:
        for section in rxn:
            for spec in section:
                if '_' not in spec and spec not in gas_spec:
                    gas_spec.append(spec)
                elif spec[-1] in site_types and spec not in surf_spec:
                    surf_spec.append(spec)
                elif not spec[-1] in site_types:
                    sys.exit(f'Error: incorrect species {spec} in reaction {rxn}')
    for spec in surf_spec:
        if '-' not in spec and '*' not in spec:
            ads_spec.append(spec)
```

```
    return gas_spec, surf_spec, ads_spec

def get_energies(energy_file, gas_spec, surf_spec):
    """Loads energies from a file and filters for relevant species."""
    energy_data = load_data(energy_file, '\t')
    g_energ = {gas: float(energy_data['formation_energy'][i]) for gas in gas_spec for i in
range(len(energy_data['species_name'])) if gas == energy_data['species_name'][i] and
energy_data['site_name'][i] == 'gas'}
    s_energ = {spec: float(energy_data['formation_energy'][i]) for spec in surf_spec for i in
range(len(energy_data['species_name'])) if spec.split('_')[0] ==
energy_data['species_name'][i] and energy_data['site_name'][i] == spec.split('_')[1]}
    return g_energ, s_energ

def shomate_eq(params, T):
    """Calculates enthalpy, entropy, and heat capacity using the Shomate equation."""
    A, B, C, D, E, F, G, H = params
    t = T / 1000.0
    H = A*t + (B/2.0)*t**2 + (C/3.0)*t**3 + (D/4.0)*t**4 - E/t + F - H
    S = A*np.log(t) + B*t + (C/2.0)*t**2 + (D/3.0)*t**3 - E/(2.0*t**2) + G
    Cp = A + B*t + C*t**2 + D*t**3 + E/(t**2)
    return H, S, Cp

def get_corrected_energies(temp, gas_spec, g_energ, shomate_params):
    """Corrects gas energies based on temperature and Shomate parameters."""
    corr_energ = {}
    for gas in gas_spec:
        temp_key = next((k for k in shomate_params[gas].keys() if temp >= float(k.split('-
')[0]) and temp <= float(k.split('-')[1])), None)
        if temp_key is None:
            sys.exit(f'Error: missing Shomate parameters for {gas} at {temp} K.')
        dH, dS, Cp = shomate_eq(shomate_params[gas][temp_key], temp)
        corr_energ[gas] = g_energ[gas] + dH  # Simplified to only use dH for correction
    return corr_energ

def calc_surf_rate_cst(T, rxn_list, corr_energ):
    """Calculates surface reaction rate constants."""
    kb = 1.380649e-23  # J/K
    h = 6.62607015e-34  # J*s
    R = 8.314462618  # J/mol*K
    k_rxn = []
    for rxn in rxn_list:
        Ea = max(corr_energ[spec] for spec in rxn)  # Assuming a single activation energy for
simplicity
        k_rxn.append(kb * T / h * np.exp(-Ea / (R * T)))
    return k_rxn

# ... other functions ...

# Main execution
if __name__ == '__main__':
    # User input for temperatures
    temperatures_C = [int(temp) for temp in input("Enter the temperatures in Celsius you want
to investigate, separated by commas: ").split(',')]

    # Loop over each temperature
    for Temp_C in temperatures_C:
        temperature = Temp_C + 273.15  # Convert to Kelvin
        dir_name = str(Temp_C)
        os.makedirs(dir_name, exist_ok=True)
        os.chdir(dir_name)
```

```
        # User input (replace with actual values or provide instructions for user input)
        catalyst = 'Pd'
        gas_fraction_dict = {'H2': X, 'C2H2': Y, 'C2H4': Z, 'C2H6': W, 'CH4': V}
        coverage_dict = {'*_s': 1.0}  # Initial coverages
        total_pressure = 1.0  # Total pressure in reactor (bar)
        sites_per_cc = a  # Number of catalyst sites per cubic cm
        vol_cstr = b  # Gas volume in CSTR (m^3)
        vol_flow_in = c # Volumetric flow into reactor (m^3/s)

        # Time settings
                t_first = 1.0e-20 # The time (s) that marks the end of the first step.
                t_final = 1.0e20 # The final time (s), after this the simulation is stopped.
                num_steps = 5 # Number of steps after which to write output.
                abs_tol = 1e-30 # Absolute tolerance of ODE solver.
                rel_tol = 1e-5 # Relative tolerance of ODE solver.
                solve_from_start = False # Solve each time point from start rather than from
                        # the previous time point


        def calc_dydt(t, act_ode):
                dens_ode = act_ode[:len(gasses)]
                cov_ode = act_ode[len(gasses):]
                pres_ode = [density_to_pressure(dens, temperature) for dens in dens_ode]
                dens_total = sum(dens_ode)

                rate_for, rate_rev = calc_rates(pres_ode, cov_ode, all_species, rate_cst,
coeff_loss, coeff_form)
                rate_rxn = [for_rate - rev_rate for for_rate, rev_rate in zip(rate_for,
rate_rev)]

                dydt = [0.]*len(all_species)
                for i in range(len(gasses)):
                        for j in range(len(rate_cst_gas)):
                                dydt[i] += coeff_form[j][i] * rate_rxn[j] - coeff_loss[j][i] *
rate_rxn[j]

                flow_correction = sum(dydt[:len(gasses)])
                for i in range(len(gasses)):
                        dydt[i] += flow_in * gas_fraction_in[i] / vol_cstr * 1.0e-6
                        dydt[i] -= (flow_in / vol_cstr * 1.0e-6 + flow_correction) *
gas_fraction_out[i]

                # Coverages
                for i in range(len(gasses), len(all_species)):
                        for j in range(len(rate_cst_gas), len(rate_cst)):
                                dydt[i] += coeff_form[j][i] * rate_rxn[j] - coeff_loss[j][i] *
rate_rxn[j]

                return dydt

        # Start simulation: loop over time intervals
    step = 0
    output_idx = 0
    t_start = 0.0
    t_step = t_first
    while t_start <= t_final:
        # Check if simulation progresses and increase t_step
        if t_step == t_start:
            t_step *= 10.0**(1.0/5.0)

        elif t_step != t_first:
```

```
        sys.exit('Simulation stuck at t = {}, simulation terminated'.format(t_start))

    if solve_from_start:
        # Solve system of ODE's
        sol = solve_ivp(calc_dydt, [0.0, t_step], activity_start, \
                        method = 'BDF', atol = abs_tol, rtol = rel_tol)
        act_out = np.transpose(sol.y)
        time = sol.t
        if not sol.success:
            print(sol.message)
    else:
        # Solve system of ODE's
        sol = solve_ivp(calc_dydt, [0.0, t_step - t_start], activity, \
                        method = 'BDF', atol = abs_tol, rtol = rel_tol)
        act_out = np.transpose(sol.y)
        time = sol.t
        for i in range(len(time)):
            time[i] += t_start
        if not sol.success:
            print(sol.message)

# Add your own writing functions as needed
```

S4. Thermodynamic data

Table S1. Enthalpies of formation [in eV] of species included in the model for the Pd catalyst with close-packed metallic facet: FCC (111). This data was calculated with respect to CH_4 formation enthalpies at 273 K and 1 bar. Rotational and vibrational wavenumbers [in cm^{-1}] of species included in the model; calculated via DFT for a (111) metallic facet of any given metal surface. Rotational wavenumbers are listed in blue.

Species	Formation enthalpy (eV) at the Pd surface	Rotational (blue) and vibrational (black) wavenumbers (cm^{-1})
H_2 (*g*)	0.09	4400.00
CH_4 (*g*)	0.00	23.55; 27.76; 33.59; 53.41; 57.90; 60.18; 1325.84; 1326.21; 1326.31; 1544.53; 1544.62; 3007.17; 3113.83; 3113.90; 3114.09
C_2H_2 (*g*)	4.41	30.92; 26.74; 0.40; 0.26; 2.08; 620.84; 620.98; 747.31; 748.50; 2056.72; 3449.37; 3551.11
C_2H_4 (*g*)	2.33	40.26; 14.03; 31.62; 42.16; 55.68; 58.66; 822.31; 942.23; 952.39; 1044.95; 1227.52; 1360.02; 1459.42; 1655.10; 3101.08; 3113.78; 3172.25; 3200.26
C_2H_6 (*g*)	0.76	12.11; 33.91; 43.50; 49.60; 50.23; 304.83; 813.70; 814.55; 981.82; 1203.26; 1203.43; 1392.72; 1400.95; 1484.23;1484.43; 1488.69; 1489.05; 2999.87; 3001.62; 3048.08; 3048.98; 3072.98; 3073.90
H*	-0.37	82.9; 85.9; 125.6
C_2H_2*	2.74	31.7; 34; 35.8; 64.2; 73.9; 93.6; 105.4; 121.8; 140.9; 161; 388.1; 390.6
C_2H_3*	2.42	20.6; 22.9; 43.1; 52.8; 53.9; 60.7; 79.7; 107.7; 119.9; 127.2; 136.5; 172.7
C_2H_4*	1.57	12.9; 14.2; 19; 45.9; 59.5; 73.9; 99.3; 112; 115.9; 124; 135; 148.9; 175.1; 179.9; 380; 381.2; 389.3; 392.4
C_2H_5*	1.31	4.6; 6.6; 7.3; 20.9; 27.6; 45.2; 68.8; 109.8; 114.7; 121.4; 137.8; 151.1; 170.6; 176.6; 180.8; 181.2; 370.1; 377.1; 379.2; 382.2; 384.6
C*	2.14	55.9; 72.5; 79.5
CH*	1.39	56.5; 57.3; 72.9; 97.7; 97.8; 383.5
CH_2*	1.37	9.4; 43.4; 64.7; 65.3; 75; 98.4; 165.6; 368.8; 378.6
CH_3*	0.68	6.6; 10.3; 10.6; 54.7; 83.2; 83.7; 141.9; 174.3; 174.9; 374.2; 387.2; 387.4
CH_3CH*	1.91	13.6; 13.7; 19.9; 30.2; 42.9; 53.4; 90.7; 116.5; 121.4; 124.3; 150.4; 169.6; 177.7; 179.2; 367.8; 372; 377.5; 380.2
H–H	0.37	82.9; 85.9; 125.6; 82.9; 85.9; 125.6

CH–H	2.16	56.5; 57.3; 72.9; 97.7; 97.8; 383.5; 82.9; 85.9; 125.6
CH_2–H	1.81	9.4; 43.4; 64.7; 65.3; 75; 98.4; 165.6; 368.8; 378.6; 82.9; 85.9; 125.6
CH_3–H	1.29	6.6; 10.3; 10.6; 54.7; 83.2; 83.7; 141.9; 174.3; 174.9; 374.2; 387.2; 387.4; 82.9; 85.9; 125.6
C_2H_2–H	3.32	31.7; 34; 35.8; 64.2; 73.9; 93.6; 105.4; 121.8; 140.9; 161; 388.1; 390.6; 82.9; 85.9; 125.6
C_2H_3–H	2.69	20.6; 22.9; 43.1; 52.8; 53.9; 60.7; 79.7; 107.7; 119.9; 127.2; 136.5; 172.7; 82.9; 85.9; 125.6
C_2H_4–H	2.05	12.9; 14.2; 19; 45.9; 59.5; 73.9; 99.3; 112; 115.9; 124; 135; 148.9; 175.1; 179.9; 380; 381.2; 389.3; 392.4; 82.9; 85.9; 125.6
C_2H_5–H	1.94	4.6; 6.6; 7.3; 20.9; 27.6; 45.2; 68.8; 109.8; 114.7; 121.4; 137.8; 151.1; 170.6; 176.6; 180.8; 181.2; 370.1; 377.1; 379.2; 382.2; 384.6; 82.9; 85.9; 125.6
CH_3–CH_2	3.49	6.6; 10.3; 10.6; 54.7; 83.2; 83.7; 141.9; 174.3; 174.9; 374.2; 387.2; 387.4; 9.4; 43.4; 64.7; 65.3; 75; 98.4; 165.6; 368.8; 378.6
CH_3–CH_3	3.65	6.6; 10.3; 10.6; 54.7; 83.2; 83.7; 141.9; 174.3; 174.9; 374.2; 387.2; 387.4; 6.6; 10.3; 10.6; 54.7; 83.2; 83.7; 141.9; 174.3; 174.9; 374.2; 387.2; 387.4
CH–CH	4.37	56.5; 57.3; 72.9; 97.7; 97.8; 383.5; 56.5; 57.3; 72.9; 97.7; 97.8; 383.5
CH_2–CH	3.89	9.4; 43.4; 64.7; 65.3; 75; 98.4; 165.6; 368.8; 378.6 56.5; 57.3; 72.9; 97.7; 97.8; 383.5
CH_2–CH_2	3.46	9.4; 43.4; 64.7; 65.3; 75; 98.4; 165.6; 368.8; 378.6; 9.4; 43.4; 64.7; 65.3; 75; 98.4; 165.6; 368.8; 378.6
H–C_2H_5	3.10	82.9; 85.9; 125.6; 4.6; 6.6; 7.3; 20.9; 27.6; 45.2; 68.8; 109.8; 114.7; 121.4; 137.8; 151.1; 170.6; 176.6; 180.8; 181.2; 370.1; 377.1; 379.2; 382.2; 384.6
H–C	2.81	82.9; 85.9; 125.6; 55.9; 72.5; 79.5

S5. Facet sensitivity analysis

To comparatively estimate the activity of the Pd (100) and Pd (211) facets and justify the Pd (111) facet choice in the model, we investigated the effects of substituting the C_2H_2 and C_2H_4 adsorption energies for those calculated for Pd (100) and Pd (211) into our microkinetic model. The model, originally developed for the Pd (111) surface, was modified by replacing only the adsorption energies for these two intermediates with values reported in the literature [1,2], due to the lack of comprehensive DFT data for all reaction species on Pd (100) and Pd (211).

Table S2. Adsorption energies (eV) of acetylene and ethylene on Pd (100) and Pd (211) surfaces, as calculated in [1]. The Pd (111) adsorption energies are also shown for comparison.

	**Pd (100)**	**Pd (211)**	**Pd (111)**
C_2H_2	- 2.66	- 2.26	- 1.94
C_2H_4	- 0.96	- 1.17	- 0.85

The results are shown in Figures S2 (a) and (b), which highlight the differences in reactivity for Pd (100) and Pd (211). Pd (100) is the least active surface, showing no C_2H_2 conversion below 600 °C, whilst Pd (211) is the most active surface, fully hydrogenating C_2H_2 to C_2H_6 at temperatures as low as 90°C.

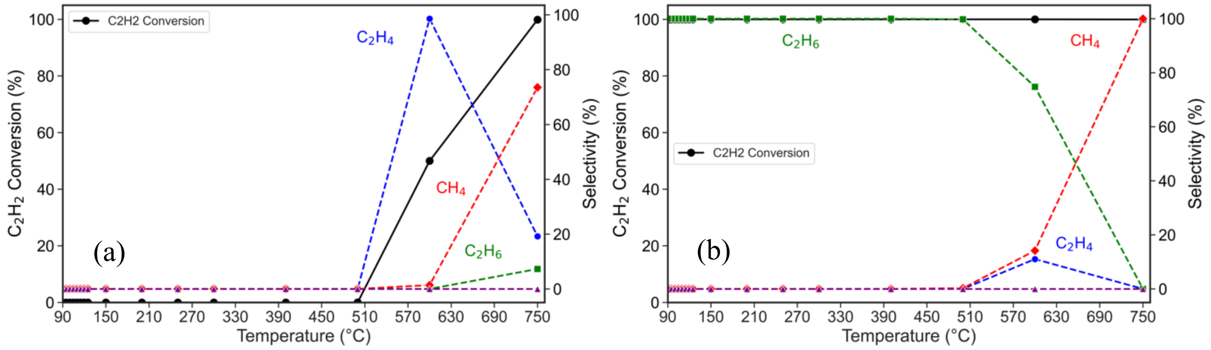

Figure S2. Calculated conversion and selectivity results for the (a) 1% Pd/Al_2O_3 catalyst with the C_2H_2 and C_2H_4 adsorption energies on the Pd (100) facet and for the (b) 0.1% Pd/Al_2O_3 catalyst on the Pd (211) facet, by replacing the C_2H_2 and C_2H_4 adsorption energies.

These findings are inherently qualitatively and do not reflect absolute activities, due to the incomplete DFT data for (100) and (211). As these results do not align with our experimental observations, this supports our choice of Pd (111) as the most representative surface for modelling the reaction conditions in this study.

S6. Modelled C_2H_2 conversion up to 750 °C

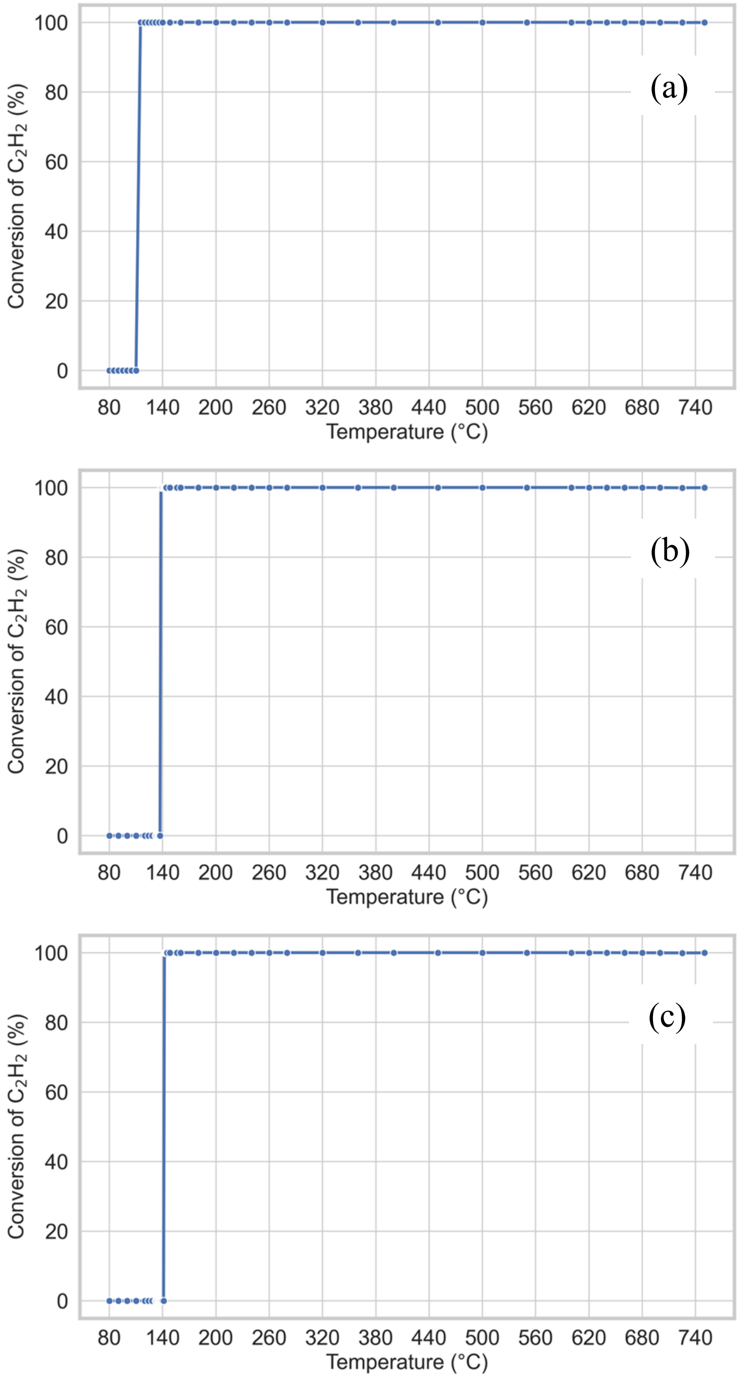

Figure S3. Modelled curves of C_2H_2 conversion as a function of temperature for the (a) 1% Pd/Al_2O_3, (b) 0.5% Pd/Al_2O_3 and (c) 0.1% Pd/Al_2O_3 catalysts. These curves show that above the activation temperature, the model predicts stable conversion at 100% for all catalysts.

S7. Time-on-stream production composition analysis

Figure S4. Time-on-stream concentration of the coupling species exiting the catalytic-plasma reactor for the (a) 0.1%, (b) 0.5%, (c) 1% Pd/Al_2O_3 catalysts.

S8. Rate analysis of $CH^* + * \rightarrow C^* + H^*$ (r_{16})

The following plot shows that the rate of the $CH^* + * \rightarrow C^* + H^*$ reaction is relatively negligible across the entire temperature range investigated. These rates are far lower than those of the CH_x hydrogenation reactions shown in Figure S11 in Section S10 below.

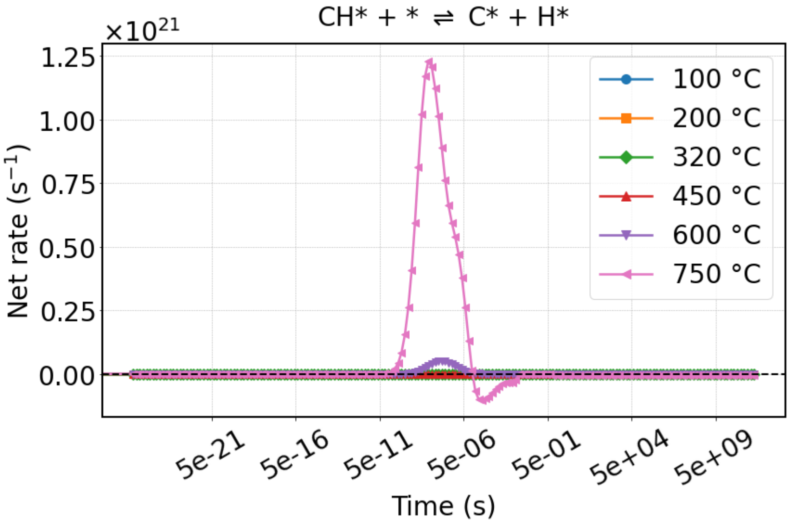

Figure S5. Net rate of the $CH^* + * \rightarrow C^* + H^*$ reaction at six temperatures covering the full studied range: 100 °C (blue), 200 °C (orange), 320 °C (green), 450 °C (red), 600 °C (purple) and 750 °C (pink).

S9. Scanning electron microscopy (SEM) of the post-reaction catalyst particles

The post-reaction catalysts were characterised by SEM as shown in Figure S6 below.

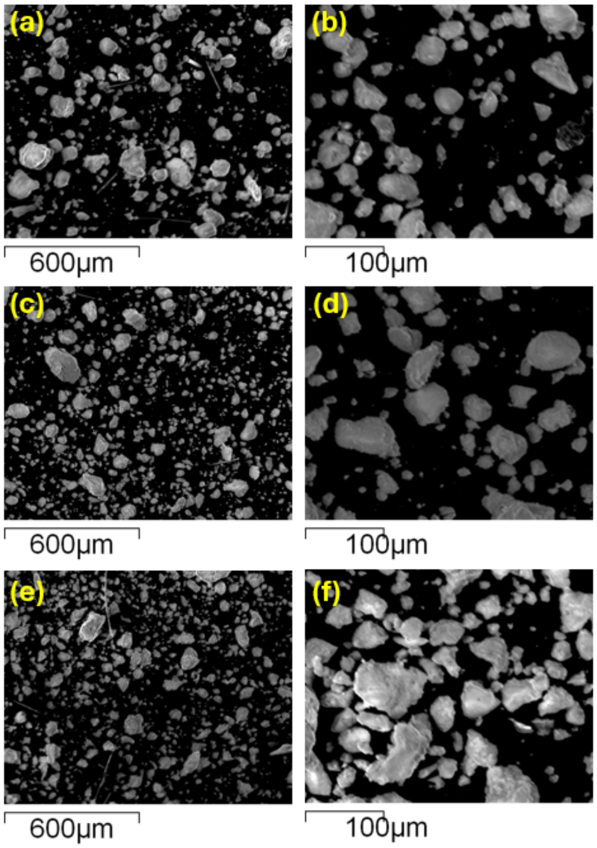

Figure S6. Scanning Electron Micrographs (SEM) of the particles collected from the catalytic bed after the plasma experiments, for the catalysts with different metal loadings: (a), (b) 0.1% Pd/Al_2O_3; (c), (d) 0.5% Pd/Al_2O_3 and (e), (f) 1% Pd/Al_2O_3.

S10. Net rates for reaction mechanism and pathway analysis

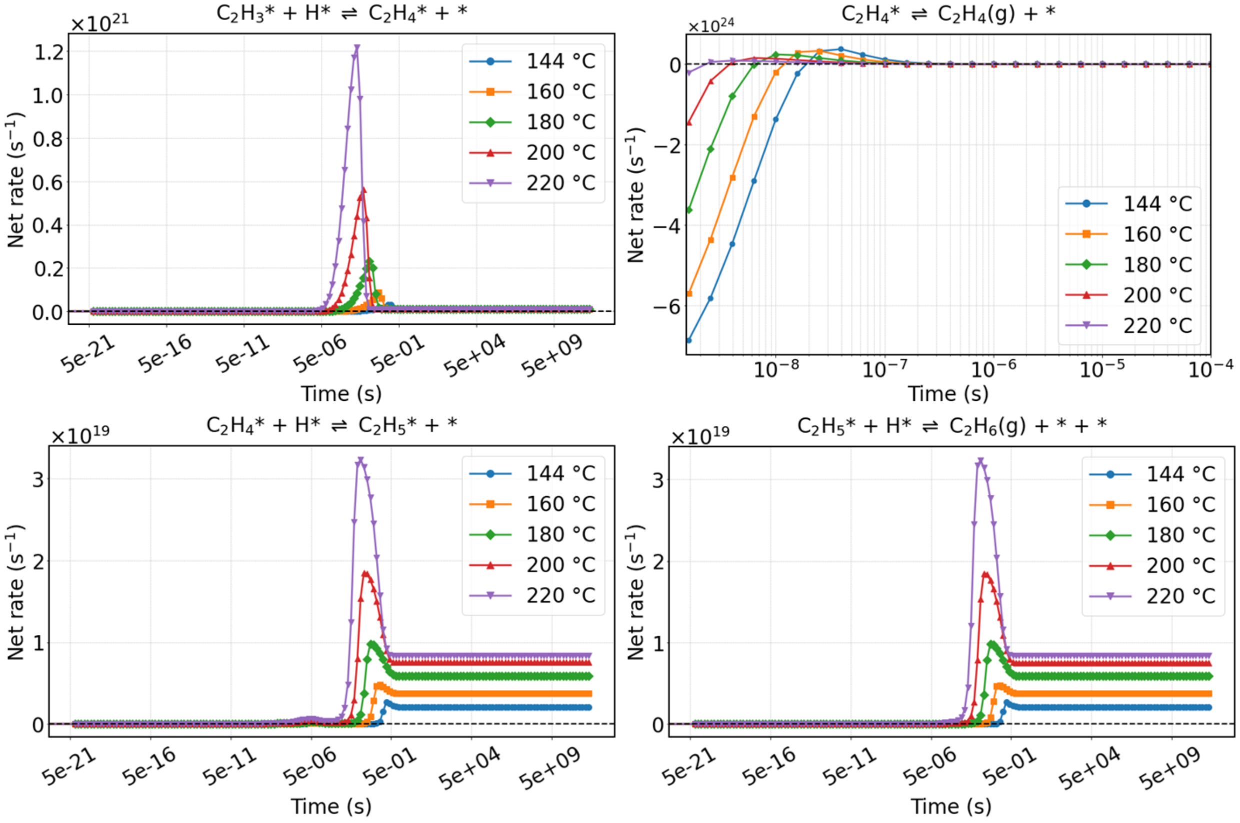

Figure S7. Net rate of selected reactions (indicated at the top of each plot) at five temperatures in the low-temperature range: 144 °C (blue), 160 °C (orange) and 180 °C (green) and 200 °C (red) and 220 °C (purple).

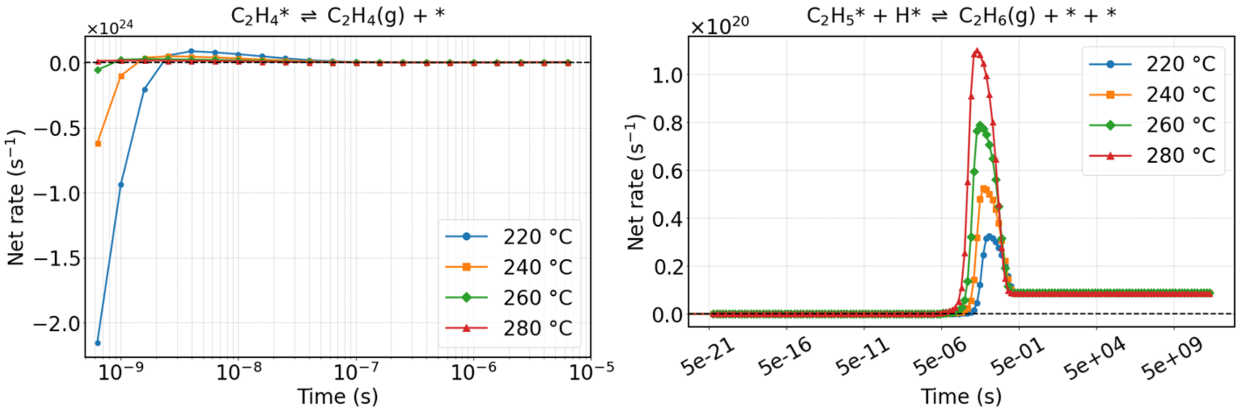

Figure S8. Net rate of selected reactions (indicated at the top of each plot) at four temperatures in the mid-temperature range: 220 °C (blue), 240 °C (orange), 260 °C (green) and 280 °C (red).

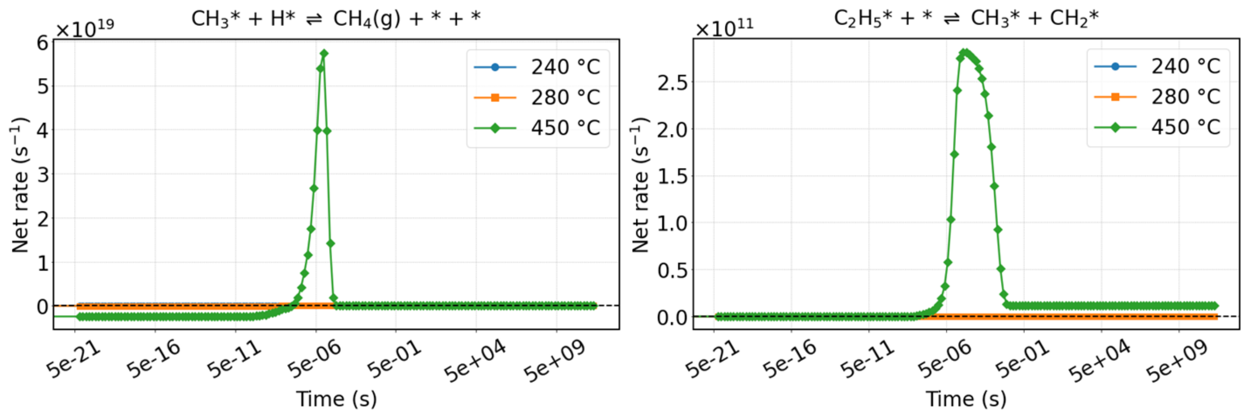

Figure S9. Net rate of selected reactions (indicated at the top of each plot) at three temperatures in the mid-temperature range: 240 °C (blue), 280 °C (orange) and 450 °C (green).

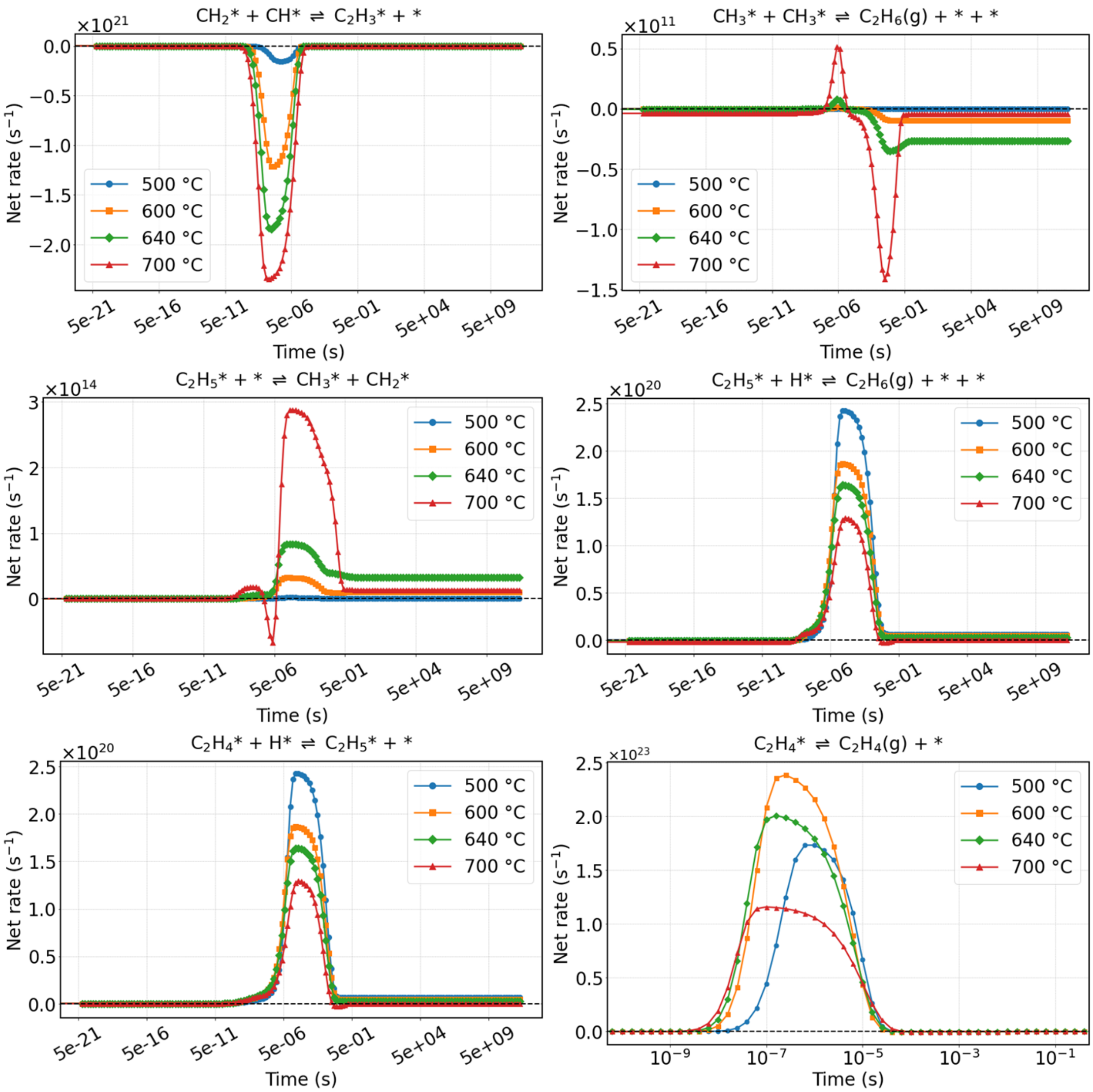

Figure S10. Net rate of selected reactions (indicated at the top of each plot) at four temperatures in the high-temperature range: 500 °C (blue), 600 °C (orange) and 640 °C (green) and 700 °C (red).

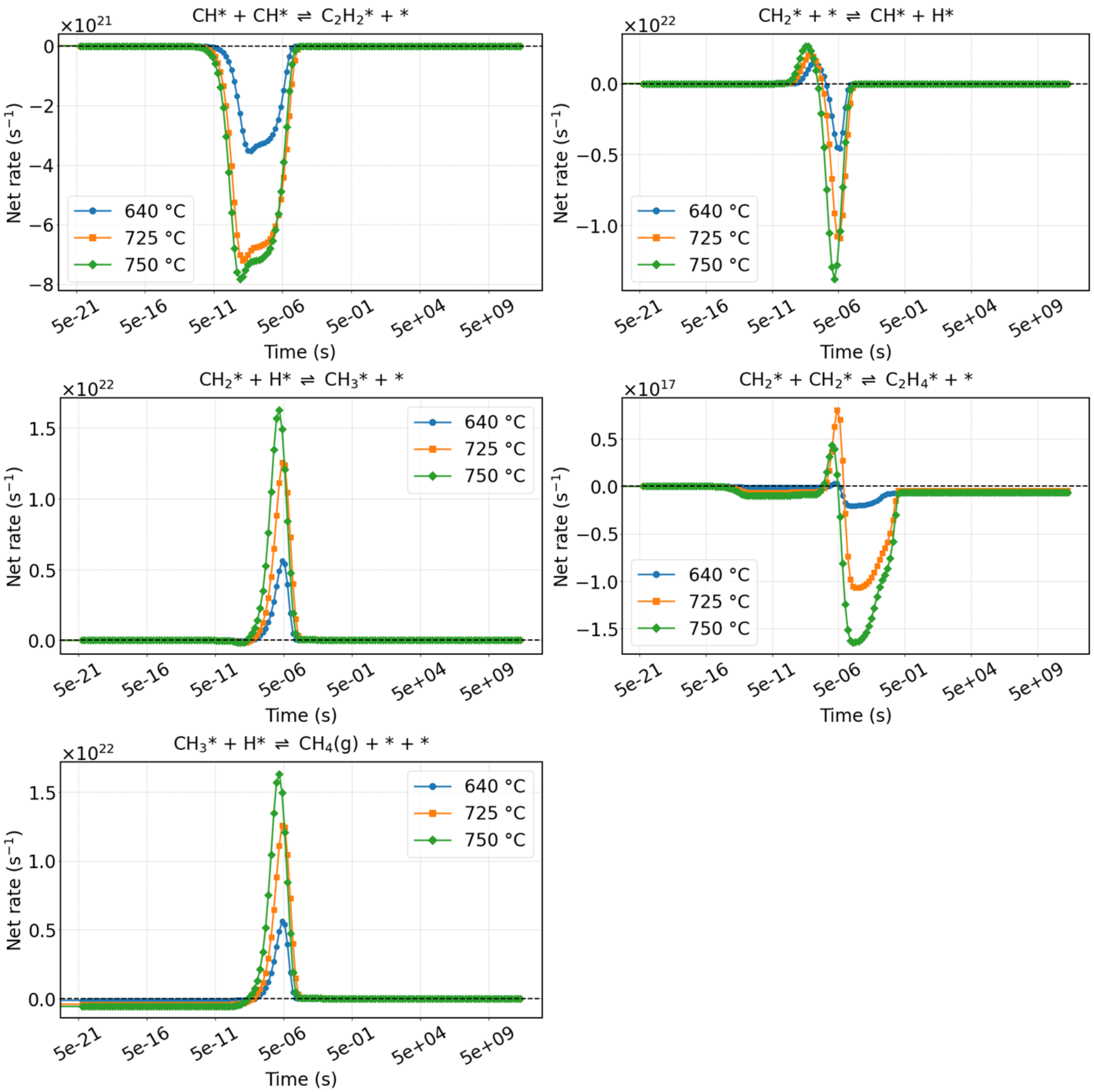

Figure S11. Net rate of selected reactions (indicated at the top of each plot) at three temperatures in the high-temperature range: 640 °C (blue), 725 °C (orange) and 750°C (green).

References

[1] B. Yang, R. Burch, C. Hardacre, G. Headdock, P. Hu, Influence of surface structures, subsurface carbon and hydrogen, and surface alloying on the activity and selectivity of acetylene hydrogenation on Pd surfaces: A density functional theory study, J. Catal. 305 (2013) 264–276. https://doi.org/10.1016/j.jcat.2013.05.027.

[2] A. Kordatos, K. Mohammed, R. Vakili, H. Manyar, A. Goguet, E. Gibson, M. Carravetta, P. Wells, C.K. Skylaris, Bridging the size gap between experiment and theory: large-scale DFT calculations on realistic sized Pd particles for acetylene hydrogenation, RSC Adv. 14 (2024) 27799–27808. https://doi.org/10.1039/d4ra03369h.
